# Reliability and Validity of Clinically Accessible Smart Glove Technologies to Measure Joint Range of Motion

**DOI:** 10.3390/s21051555

**Published:** 2021-02-24

**Authors:** Jeffrey Henderson, Joan Condell, James Connolly, Daniel Kelly, Kevin Curran

**Affiliations:** 1 School of Computing, Engineering and Intelligent System, Ulster University Magee Campus, Northland Rd, BT48 7JL Londonderry, Ireland; j.condell@ulster.ac.uk (J.C.); d.kelly@ulster.ac.uk (D.K.); kj.curran@ulster.ac.uk (K.C.); 2 School of Science, Letterkenny Institute of Technology, Port Rd, Gortlee, F92 FC93 Letterkenny, Ireland; james.connolly@lyit.ie

**Keywords:** rheumatoid arthritis, Parkinson’s disease, stroke recovery, smart sensing, data gloves, joint measurement, rehabilitation, measurement system

## Abstract

Capturing hand motions for hand function evaluations is essential in the medical field. For many allied health professionals, measuring joint range of motion (ROM) is an important skill. While the universal goniometer (UG) is the most used clinical tool for measuring joint ROM, developments in current sensor technology are providing clinicians with more measurement possibilities than ever. For rehabilitation and manual dexterity evaluations, different data gloves have been developed. However, the reliability and validity of sensor technologies when used within a smart device remain somewhat unclear. This study proposes a novel electronically controlled sensor monitoring system (ECSMS) to obtain the static and dynamic parameters of various sensor technologies for both data gloves and individual sensor evaluation. Similarly, the ECSMS was designed to closely mimic a human finger joint, to have total control over the joint, and to have an exceptionally high precision. In addition, the ECSMS device can closely mimic the movements of the finger from hyperextension to a maximum ROM beyond any person’s finger joint. Due to the modular design, the ECSMS’s sensor monitoring board is independent and extensible to include various technologies for examination. Additionally, by putting these sensory devices through multiple tests, the system accurately measures the characteristics of any rotary/linear sensor in and out of a glove. Moreover, the ECSMS tracks the movement of all types of sensors with respect to the angle values of finger joints. In order to demonstrate the effectiveness of sensory devices, the ECSMS was first validated against a recognised secondary device with an accuracy and resolution of 0.1°. Once validated, the system simultaneously determines real angles alongside the hand monitoring device or sensor. Due to its unique design, the system is independent of the gloves/sensors that were tested and can be used as a gold standard to realise more medical equipment/applications in the future. Consequently, this design greatly enhances testing measures within research contact and even non-contact systems. In conclusion, the ECSMS will benefit in the design of data glove technologies in the future because it provides crucial evidence of sensor characteristics. Similarly, this design greatly enhances the stability and maintainability of sensor assessments by eliminating unwanted errors. These findings provide ample evidence for clinicians to support the use of sensory devices that can calculate joint motion in place of goniometers.

## 1. Introduction

In medical applications, such as rehabilitation and hand function assessment, capturing hand kinematics is necessary [1]. Rheumatoid arthritis (RA), Parkinson’s disease (PD), and other neurological conditions have a detrimental effect on the quality of life of the affected people [2]. RA is an inflammatory condition characterised by the discomfort, stiffness, swelling, and deformity of affected joints [3]. Drug medications and therapies are directed at reducing the debilitating effects of RA, preventing disease growth, and bringing the disease into remission [4]. Furthermore, the early diagnosis of RA is essential for the initiation of treatment [5]. Similarly, joint stiffness is a common complaint of RA sufferers and is one of the first signs [6]. However, its unpredictability amongst patients and its measuring difficulties have diminished the value of joint stiffness as an RA identifier [3].

Clinicians currently assess a patient’s joint movement using a universal goniometer (UG) device to determine joint restriction [7]. However, when measuring static joint range of motion (ROM), the UG is not without its disadvantages [8]. Likewise, there may always be some degree of error due to the device not typically being practical enough to be aligned directly with the appropriate landmarks on the metacarpophalangeal (MCP) joints, the proximal interphalangeal (PIP) joints and the distal interphalangeal (DIP) joints of the fingers [9]. Some factors such as the location of the device and the therapist’s own technique in the interpolation of anatomical landmarks, can cause the lack of consistency when using a UG [8]. Therefore, it is no surprise that reliability is reduced when measuring each small joint independently because the human hand comprises 27 bones of approximately 25 degrees of freedom (DOF) [2]. Other neurological disorders like PD usually begin with a tremor in one hand but can also cause stiff limbs or slow motion without tremors, thus making the onset of the disease difficult to diagnose at the early stages [10].

Capturing accurate hand kinematics is necessary so that clinicians can reliably assist with the diagnosis of RA and PD, as well as rehabilitation activities, including the recovery of stroke patients [11]. Consequently, there is a need for an ambulatory system capable of assessing regular improvements in patient activity to recognise the symptom of joint stiffness [3]. To clarify, a practical and reliable device that can record several parameters of hand motion to assist with disease diagnosis and in the field of rehabilitation is required [11]. Unknown physiological effects and the reduced repeatability of findings have constrained the use of devices in clinical practice. To summarise, wearable technology should be capable of continuously monitoring the hands without causing obstruction to the daily tasks of the patients whilst also providing the clinician with precise feedback [12].

The purpose of this paper was to analyse the accuracy, repeatability, and linearity of resistant transducers (resistive bend sensors), fibre optic bend sensors, inertia measurement units (IMUs), and Hall effect sensors used for medical applications and to provide unfamiliar, detailed, and reliable facts for the clinical area of smart wearable systems/smart gloves. Furthermore, this paper aimed to include an in-depth survey report of glove-based sensor technologies for medical applications to remove the foregoing drawbacks of previous studies. Throughout the last few decades, a high number of research papers and studies of prototype devices have been published. However, a low level of demonstration of such sensors has been examined in the field of instrumented gloves applied for rehabilitation and assistive technologies. Up to this point, review papers have been very basic, focusing on one technology with little detail on sensor characteristics during dynamic movements.

This paper is organised into six main sections. After the introduction, Section 2 provides an overview of the relevant literature. Section 3 leads into the implementation of a novel electronically controlled sensor measurement system (ECSMS). Other unvalidated measurement devices have been constructed by research teams to evaluate the characteristics of sensors, but they have been limited in design and have not corresponded to that of dynamic finger joint movement. Likewise, this system proposes to finalise the unknown physiological effects and reduced repeatability of findings constrained with the use of sensory smart glove devices. Section 4 covers specially developed testing protocols/testing strategies to analyse various data glove sensors for precision and reliability, as well as the ECSMS itself. Furthermore, Section 5 focuses on the level of accuracy of various sensor technologies used in data gloves. Similarly, the final section focuses on the level of repeatability of various sensor technologies that have been widely researched throughout the years.

In conclusion, this paper focuses on the development of an objective system capable of monitoring any contact or non-contact system with a high precision. The final sensory data extracted from the novel ECSMS will determine what sensors have the potential to monitor finger joint movement to assess and detect changes in ROM and flexibility that identify the characteristics of joint stiffness. Furthermore, this research details a data glove/sensor measuring device that is required for an objective measurement system. Equally, a reliable data glove device is required to assess joint mobility, as it avoids typical intertester reliability issues associated with clinical evaluation approaches such as goniometric calculation and visual inspection. This paper will assist clinical practitioners and researchers to finally verify the correctness and choice of sensor technology for a clinical glove device.

## 2. Accuracy, Repeatability, and Resolution

Clinicians tend to favour IMUs, as they can measure a variety of factors, including acceleration, force, angular rate, and direction in the presence of a magnetometer [13]. However, IMUs can be affected by accumulated errors and other environmental factors [7]. Other factors such as cost, size, measurement function ability, and physical structure can all play a part into the selection of sensors for a wearable device [2]. Furthermore, researchers and clinicians seek the next generation wireless smart glove but are often overcome by issues relating to the linearity of sensors [2,14]. Two important testing strategies for linearity are to assess the accuracy and to assess repeatability of a sensor during angular movement [3]. Consequently, these tests can also display the characteristics of the sensor during angular movement.

Previous researchers have created sensor testing devices to measure the accuracy, repeatability, and linearity of a sensor. However, these objects and custom-made devices were not able to extract static data, not to mention dynamic measurements, to a reasonable level. This suggests that an ineffective sensor/glove measurement system may allow for false data to be accepted and good data to be rejected, resulting in wasted time and money, as well as the uncertainly of the technologies characteristics.

Connolly [3] used various wooden blocks cut to known angles to measure angular data of sensors within data glove devices. As a result, this had a huge disadvantage of only providing static and course angle verification. Lin et al. [1,15] developed two self-made angle measurement devices, with the first device used to validate the static angle of an IMU. This device was limited to rough static movement, and the measuring device had to be observed at each angle whilst reading a basic mathematical protractor. The second device was specified to have performed dynamic measurement on the IMUs. However, this was not possible to a high degree because the measuring device had no factual dynamic output against the IMU being examined. Unlike the ECSMS in this paper, these devices were not made for precise movements with their low-precision hinging mechanism that operated via a servo motor rather than a stepper motor. Besides, the sensor output was not being streamed simultaneously against a dynamic and validated device to match that of real finger joint movement. However well these devices perform for IMU investigations, the ECSMS mainly focuses on how all sensors perform in and out of a glove to a very high level of precision that follows real hand joint movement.

Different techniques from measurement system analysis (MSA) are used to evaluate the performance of a measurement system/device [16]. Various methods evaluate each aspect of the process, including the test protocol, any measuring instruments (secondary equipment), and the measurement techniques used. The first step defines whether the correct secondary measurement device is selected to validate the measuring system. The second step defines an appropriate protocol to validate the sensor measurement system. Subsequently, the third and final step defines a protocol for testing the sensors using the validated measurement system. Moreover, the measurement device/sensor data being measured and the methods and instruments used to collect and document the data are carefully reviewed [17].

Initially, the aim was to quantify the effectiveness of the ECSMS, analyse the variation in the data, and then repeat the exact same procedure on other sensors using the validated contact system. This ensured that the collected data was trustworthy and that the ECSMS was in fact suitable as a measurement device itself. In addition, for the design process, good reliable data could prevent wasted time regarding wearable technologies. The collected data were evaluated for bias, stability, and linearity. Utilising MSA techniques, the amount of measurement uncertainty of the ECSMS was initially reduced. The system was found to have a precise level of resolution to obtain data that that one would expect of a measurement system. To summarise, the ECSMS was assessed in terms of precision, resolution, accuracy, repeatability, and linearity before any sensor technologies were evaluated. Listed below are the key techniques/tests and observations.

Accuracy is the degree of conformity of a measured quantity to the actual position measured by the measurement device [18,19].

Repeatability is characterised as the range of positions reached under similar conditions (e.g., the device is repeatedly instructed to one location multiple times) [20]. Additionally, for repeatability to be measured, the following conditions must be met; use the same observer/instrument, same measurement process, and same location under the same conditions/same repetition over a short period of time. The “repeatability coefficient” is a precision statistic that signifies an absolute inconsistency between a pair of replicated test results [18,19].

Precision is the degree to which additional measurements show the same or comparable results [18].

Linearity is the change in bias over the operating range of the measurement equipment. Likewise, the linearity of a sensor/device is an expression of the extent to which the actual measured curve of the sensor deviates from the ideal linear slope [14,19].

Resolution is the smallest measurable gradual shift in the input parameter to be observed on the output signal of a sensor or device. Moreover, resolution can be conveyed as either a percentage of reading (or a full-scale reading) or in absolute terms [19].

Many researchers have focussed on testing smart glove devices as a final working item. In this paper, each sensor technology was examined extensively using a novel sensor measurement device. Current glove systems were stripped back to show the true characteristics of various technologies. In addition, the high-precision measurement system was found to closely model a human finger joint and was the first of its kind in terms of smart glove monitoring. Moreover, no textile materials or intrusive exoskeleton devices could get in the way with the true characteristics of each sensor exposed. All sensors were made to be contained in unique capsules/pockets like they are in data gloves, though with reduced interventions. This prevents any unwanted interferences or discrepancies between tests (e.g., unwanted stresses, frictional forces, and pressures).

## 3. Design of Sensor Monitoring System (ECSMS)

A high-precision sensor measurement/monitoring system was designed to examine all smart glove technologies. The system currently measures sensors on their own with no textile materials that may cause obstructions. In addition, the device was designed while keeping any future additional modifications in mind (e.g., there are plans to advance the system in the nearer future to add a textured surface in order to model a real finger). Consequently, this verified whether any differences occur when using sensors on their own whilst examining sensors on the ECSMS. Moreover, with this design in mind, the system has the capabilities of experimenting with different sensor technologies for all kinds of fabrics (e.g., different glove materials may be placed over the end of a rubber/textured finger). Consequently, this design benefits the sensor selection process while also allowing for the material and the material pockets/holders to be adopted using this system in the future.

### Overview of Data Glove Testing System

**Figure 1:**
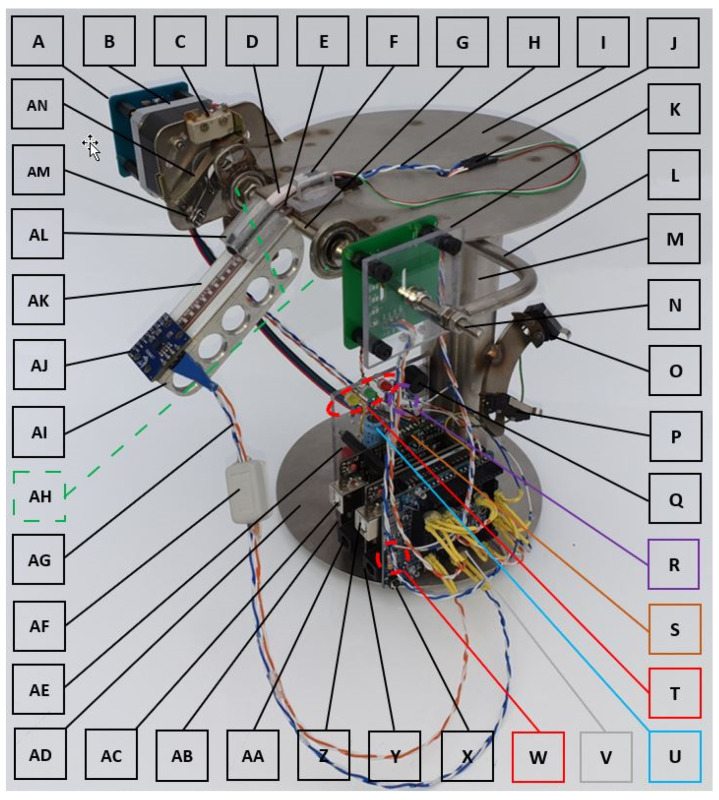
Electronically controlled sensor measurement system (ECSMS) design and its components.

The ECSMS was developed as a tool for capturing sensor motion during static and dynamic movements. It contains multiple microcontrollers, a moving arm (to be advanced), and a programmable system. The detailed design of the device is shown in Figure 1. Ultimately, this system brings huge potential in the testing of glove systems, where the monitoring high-precision sensor can cover every angle accurately from hyper extension (minimum sensing range of −15°) to maximum flexion (maximum sensing range of 130°). In other words, the dynamic arm is much like an electronically controlled finger, having the capability of capturing movements in real time whilst monitoring other glove technologies. In addition, with any sensor to be tested attached, each sensor can move freely within the ECSMS’s range and accordingly with the controllable software program. The main objective is to position the sensors accurately and to control their movement whilst monitoring them with a high level of precision. To summarise, the data from all the outputs can be gathered to determine the real/true characteristics of sensor technologies.

Table 1 shows a list of all the custom manufactured parts and general components along with the various testing sensors used throughout this paper. The explanation that follows demonstrates the high level of precision the system was designed too. The ECSMS was designed and manufactured using a high grade 316 stainless steel (**I**, **L**, **M**, and **AD**). The non-ferrous material, aluminium, was used for the moving arm (**AI**) to reduce the presence of ferromagnetic materials to ensure that there was no unintentional magnetic interference on the sensors, such as IMUs. Furthermore, the system was made to incorporate two Arduino microcontrollers, a sensor board (**Z**) (Arduino 1), and a stepper motor controller board (**AC**) (Arduino 2) that controls a stepper motor (**B**). Moreover, both microcontrollers have 14 digital input/output pins and 6 analogue pins that were required for multiple sensor inputs and multiple outputs for the stepper driver.

The ECSMS’s homing circuit, the stepper motor driver circuit, and the high-precision encoder (**K**) are controlled by one Arduino (**AC**). In addition, the stepper motor can move in increments of 1.8° at 200 steps per revolution whilst being stepped by the microcontroller. However, this is not fine enough to achieve the desired fine resolution (0.1°) of the secondary device the system was being validated against. A micro step driver (**S**) was required to reduce the size of the steps to 1/2, 1/4, 1/8, 1/16, 1/32, 1/128, and 1/256 micro steps. As a result, this reduced the movements to a minimum of 1/256 micro steps or 0.007° per step. To clarify, this was designed to provide very small incremental movements, bringing the level of resolution and precision to a much higher degree than the secondary device, or any IMUs, or similar digital components. Furthermore, it is important to note that the system could only be validated to the secondary device’s accuracy of 0.1°. However, this is an exceptional level of accuracy for testing sensors, where a higher accuracy provides more detailed sensor characteristics during testing.

A Flexpoint sensor (**E**) slides freely into a small (capsule) machined recess (**AK**), with a small piece of polycarbonate keeping the sensor in place. This capsule modelled the pockets of the ActionSense glove that uses multiple Flexpoint sensors. The analogue (flex/bend) sensor is connected to a voltage divider circuit (**R**), whilst its output is connected to the analogue input of the sensor board (**Z**). Furthermore, a fibre optic sensor (**D**) also slides freely in and out of a machine precision polycarbonate capsule (**AL**). This capsule was made to be of identical size to the Fifth Dimension Technologies (5DT) glove’s capsule/pocket to match the sensor’s face area. However, for accurate measuring, the 5DT sensor has a small piece of Vaseline placed inside the capsule to remove stresses/friction on the Bragg grating/fibre core whilst it is being worked by the ECSMS. Moreover, the fibre optic sensor also has its own circuit (**W**) to provide power (1.2 V) for the infrared (IR) light emitting diode (LED) and for the photodiode’s altering control signal to the sensor board (**Z**). It is important to note both the ActionSense’s Flexpoint sensor and the 5DT’s sensor are held in position (**F**) at one end, just as they are in the glove.

Other sensors such as IMU (**AJ**), Hall effect (**A**), and capacitive sensors (**none attached**) can all be precisely monitored throughout the ECSMS’s range. These digital sensors are directly wired to the sensor board’s (**Z**) digital pins. Furthermore, a custom jumper board (**V**) allows the sensors to be interchanged during tests or to connect additional sensors (e.g., capacitive).

Ferrite clamps (**AF**) and decoupling capacitors (**U**) are used to remove electromagnetic interference (EMI) produced from the high current stepper driver circuit that could influence the sensor program (added shielding).

The moving arm (**AI**) is spot-welded to the custom main shaft (**G**), where the shaft is monitored by a highly precision encoder (**K**). In addition, the encoder is attached to the main shaft with the capability of adjustment at point (**N**) to set the sensor to zero position with respect to the arm (**AI**). Furthermore, it is important to note this linear encoder is independent of the stepper motor and uses the inter-integrated circuit (I2C) serial communication protocol.

A stepper motor (**B**) is coupled to the main shaft held with 2 small precision roller bearings (**AH**) to reduce any friction/drag on all the components.

It is significant that the stepper motor is aware of the starting position before the initiation of each test. For this reason, a switch lever (**AN**), a moving arm (**AM**), and a microswitch (**C**) all work together in an accurate homing circuit designed to ensure that the device is automatically fine-tuned to the reference location/zero before the ECSMS begins. Furthermore, the measuring system returns to reference zero between each repeatability test and before it begins to work its way through the complete (pivoting arm) finger range during accuracy tests. In fact, reference zero (home position) is always in the range (0.007°) of the stepper driver and within the level of accuracy of the encoder (0.0087°).

A beeper (**Q**) circuit was set up for the ECSMS’s validation process, where a short pulse is heard when the ECSMS stops at each position, signifying the user to take readings of the secondary device. Hence, this only needs to be performed once during the validation process of the ECSMS.

The USB connector (**Z**) outputs all the sensor data alongside the high-precision encoder of the ECSMS. A driver directs the stepper motor to pre-set angles, where the controlling personalised accuracy and repeatability programs are uploaded via the USB connector (**AC**).

The amber indicator (**T**) denotes that the homing cycle is in operation (always less than 1 s), the green indicator denotes that the system is running, and the red indicator denotes that the system has stopped. It is important to note that the ECSMS never finishes a test cycle at reference zero. Hence, the system moves to approximately 30° (downhill) away from reference zero for the next homing cycle/test to execute. This ensures a high level of accuracy between measurements.

To summarise, switch B (**O**) instructs the ECSMS to follow a dynamic movement depending on the upload (**AC**) (repeatability or accuracy test). However, before that, the system finds reference zero and automatically starts the calibration process (e.g., accelerometers, gyroscopes, and magnetometers). Furthermore, when all the indicators are flashing together in a moving sequence, this signifies that the ECSMS has found reference zero, triggering the calibration process. Moreover, switch B only becomes activated after calibrations are complete. Switch A (**P**) is optional and can also be used to trigger the sensor board’s calibration process (**Z**). Note: When the desired range is met/record point, a ‘low’ signal from board (**AC**) currently alerts the sensor board (**Z**). Finally, at this time, sensor information/data are all simultaneously streamed (on screen serial monitor/serial graph or COM Port (Serial Port)/Tera Term) against the validated ECSMS depending on the devices connected. Please see Supplementary Materials (ECSMS demo, Video S1).

## 4. ECSMS Validation Process Overview

The initial step in the validation process was to prepare a protocol to assess the accuracy and repeatability of the novel ECSMS. Both accuracy and repeatability tests were used as acceptance criteria referring to the earlier literature on MSA techniques. Furthermore, the linearity, stability, resolution, and precision of the ECSMS and sensors were observed throughout the range. The precision of the ECSMS could be expressed as a percentage of the full-scale value or as an absolute value. Moreover, relevant statistical techniques were used to determine if any two sets of values had any kind of relationship or if the results of all experiments were statistically significant.

The ECSMS was measured against a secondary device [21] to determine the devices’ errors throughout the range, i.e., the error between the actual value of the secondary device and the indicated value on the output of the ECSMS. First, the secondary device (inclinometer) was mounted to the pivoting arm of the ECSMS, where a thin layer of adhesive tape was used to attach the device firmly onto the ECSMS’s arm. Second, the simple protocol in Section 4.1 was followed to determine if the mean of both sets of data for one collected sample resembled the validated and calibrated measurement of the secondary device. Likewise, the ECSMS’s accuracy was defined as the extent to which a specified measurement from the secondary device (0.1°) agreed with the definition of the quantity that was being measured.

A pre-set accuracy program code was uploaded to the ECSMS, where the stepper driver was set into 1/256 micro stepping mode. This consequently produced fine movement of the stepper motor that was required to match the resolution of the secondary device. The ECSMS was then stepped swiftly from 0° to 125° in 0.1° increments at a rate of 0.1°/a second. Furthermore, this simultaneously generated 2 sets of data, streaming 2468 values in total. Once the accuracy test was accomplished, results were analysed to determine the level of agreement between the ECSMS and the secondary device. Similarly, during repeatability testing, a variation of repeated measurements was completed with the same observer and the same equipment, all under the same conditions over a short period of time. A pre-set programming code was uploaded to the ECSMS, where the system recorded in 20 intervals (n = 20) at every 5° throughout the ECSMS range (0–125°). Moreover, this also simultaneously generated 2 sets of data, producing nearly 1100 values in total between the measurement system and the secondary device.

### 4.1. Testing Protocol

A two-part protocol test method was set to validate the ECSMS and to test all the analogue and digital sensors. During this process, the system followed the resolution (0.1°) and accuracy (0.1°) of the secondary device. Throughout the ECSMS’s validation process, only the parameters of groups 1a and 1b were executed. During the sensor’s accuracy evaluation procedure, only the parameters of group 2a were executed. Likewise, throughout each sensor’s repeatability evaluation process, only the parameters of group 2b were executed.

Group 1a: ECSMS accuracy testing (validation accuracy test only completed once) (Section 4.2).

1. The system stepped from parameters 0° to 125° in 0.1° increments, recording the ECSMS’s arm at each position.
2. Data were observed and recorded of the secondary device during the count intervals (only needed to be done once).
3. Statistical methods (ANOVA, Pearson’s correlation, and Bland–Altman charts) were used to show the level of agreement.

Group 1b: ECSMS repeatability testing (validation repeatability test only completed once) (Section 4.2).

1. The system stepped from parameters 0° to 125° in 5° increments, recording the ECSMS’s arm at each position.
2. Data were observed and recorded of the secondary device during the count intervals (only needed to be done once).
3. Statistical methods (ANOVA, Pearson’s correlation, and Bland–Altman charts) were used to show the level of agreement.

Group 2a: Glove/sensor accuracy testing (Section 5).

1. The system stepped from parameters 0° to 125° in 0.1° increments, recording the ECSMS’s arm at each position.
2. Data glove/sensor data were recorded simultaneously alongside the ECSMS during the count intervals.
3. Statistical methods (ANOVA, Pearson’s correlation, and Bland–Altman charts) were used to show the level of agreement.

Group 2b: Glove/sensor repeatability testing (Section 6).

1. The system stepped from parameters 0° to 120° in 5° increments, recording the ECSMS’s arm at each position.
2. Data glove/sensor data were recorded simultaneously alongside the ECSMS during the count intervals.
3. Statistical methods (ANOVA, Pearson’s correlation, and Bland–Altman charts) were used to show the level of agreement.

The statistical considerations and analytical plan are as follows.

Aims and Endpoints:

Objective 1: Level of agreement between the ECSMS and secondary device.

Objective 2: Measurement of error when using the validated ECSMS to evaluate the accuracy and repeatability of each data glove/sensor (assessing the linear relationship of both devices simultaneously).

### 4.2. Validation of ECSMS

Throughout this research, descriptive and statistical methods were used to analyse the accuracy/repeatability between the ECSMS and data glove sensors (i.e., ANOVA, Pearson’s correlation, and Bland–Altman charts). Outside variables, such as room temperature, were excluded from testing procedures. The testing procedure followed the protocol shown in Section 4.1 (groups 1a and 1b) Findings from the ECSMS were divided into two categories.

**Figure 2:**
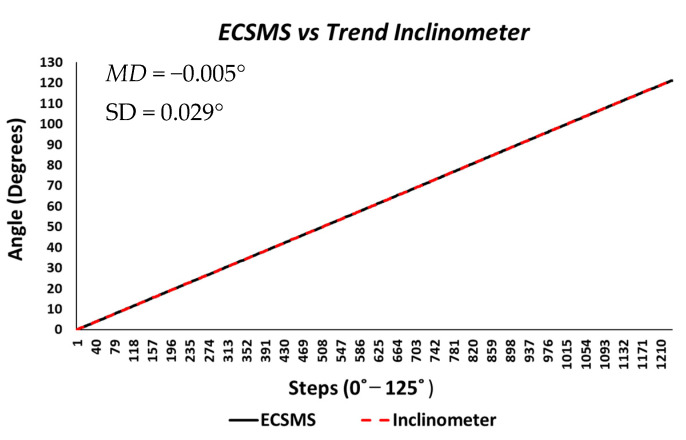
Accuracy test—electronically controlled sensor testing system (ECSMS) vs. calibrated secondary device (Inclinometer). MD: mean difference; SD: standard deviation.

**ECSMS accuracy testing:** the ECSMS and secondary device were both measured continuously throughout the range in order to mimic how the sensors performed in a data glove. The secondary device was used as an independent reference to validate the angular accuracy of the ECSMS. Furthermore, the ECSMS prompted the user to take the reading of the device when a short pulse beep was outputted. Figure 2 shows both the ECSMS’s output and the inclinometer’s output where a perfect linear response was seen. To clarify, the horizontal axis defines the number of steps taken to get to the maximum angle (125°), while the vertical axis highlights the specific angle completed in degrees.

The count required of both devices to get to 125° was 1234 steps. The accuracy test generated 2468 values in total, all performed under the same conditions at the same time. The average and variance values between the devices were near perfect, as presented in Table 2. Furthermore, descriptive statistics were calculated for both devices. As a result, the standard error of the mean differences and the Standard Deviation (SD) of the mean of the differences were considerably low (Mean Difference (MD) = −0.005°; SD = 0.029°). Moreover, the differences suggested that the ECSMS was very capable of maintaining the 0.1° accuracy and resolution of the secondary device. Pearson’s correlation is a good measure of simultaneous reliability tests and was calculated for comparison with earlier research studies. ANOVA and a Pearson correlation coefficient test were performed (also presented in Table 2) to identify any significant associations between the two devices. In this experiment, a *p*-value of less than 0.05 (typically ≤0.05) was considered statistically significant. However, a *p*-value of nearly 1 signified that the two groups were near identical. We accepted the null hypothesis $H_{O}:\mu_{1}=\mu_{2}=\mu_{k}$ because there is no significant difference between the ECSMS and the secondary device during the accuracy test. There was a positive correlation (r = 1), thus indicating a perfect positive relationship. Both sets of data moved in the same direction together.

**Figure 3:**
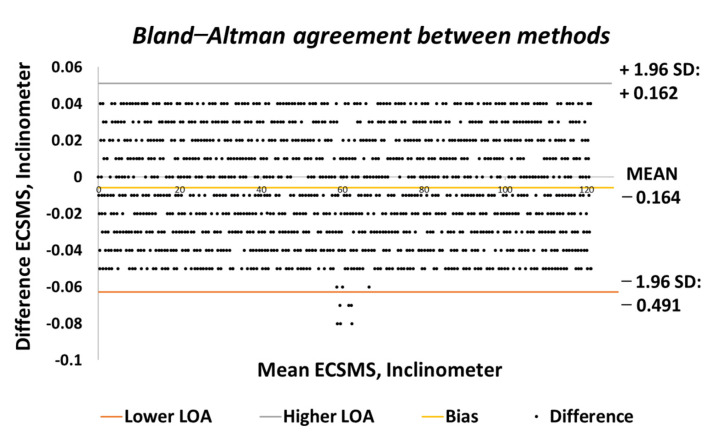
Accuracy test—agreement between ECSMS and secondary device. LOA: limit of agreement.

Figure 3 shows the 95% confidence interval (CI) on a Bland–Altman chart, where a low variability resulted in a narrow CI with a small margin of error (approximate lower limit of agreement (LOA) = −0.06° and upper LOA = 0.05°).

**Figure 4:**
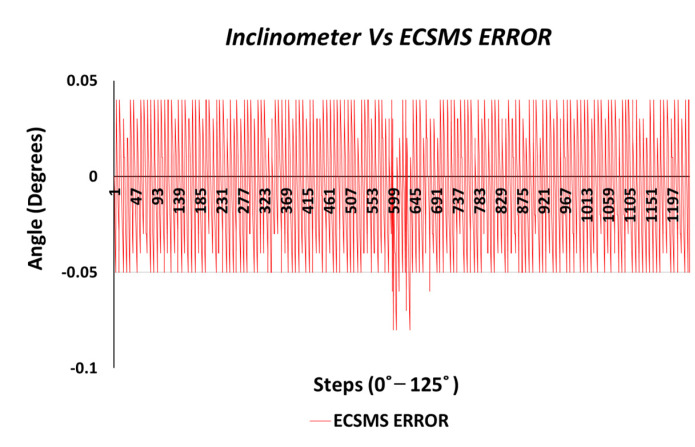
Accuracy test—ECSMS error vs. secondary device.

Similarly, the correlation was strong, giving a high level of agreement between the devices. The trend was highly repetitive and steady with few discrepancies (outliers) at midrange (45°) bending. In addition, the small number of outliers could have been the cause of measurement variability, or they may have indicated small experimental errors, but it is more than likely that it indicated a small error of the secondary device at mid-way bending. Nevertheless, this did not disadvantage the 0.1° accuracy, as demonstrated in Figure 4 because the values were well inside the lower limit.

**ECSMS repeatability testing:** this section compares the repeatability results of both devices using various statistical methods. Performing this test examined the capability of the ECSMS against the secondary device to consistently replicate angular readings when put through a dynamic testing strategy. Presented in the Table 3 is a summary of the test results extracted from the ECSMS. For instance, the thorough test consisted of one main test segmented into 26 smaller tests containing 20 samples (n = 20). Similarly, in between each sample, the ECSMS returned to zero and then back to the testing angle at a maximum of 20 times. Moreover, once the ECSMS had finished one minor test in the group sample, the system performed a homing cycle to set the ECSMS back to reference zero for the next set of tests. Hence, this sequence was set to reset the system at every 20 steps to reduce the chances of the ECSMS running off over the 520 positions (major test).

**Figure 5:**
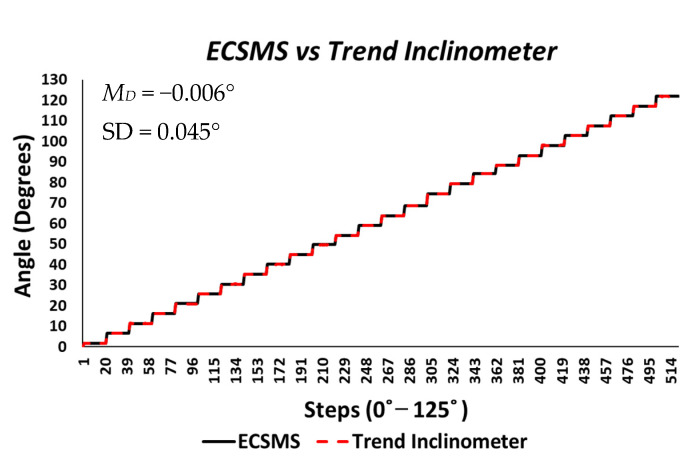
Repeatability test—electronically controlled sensor testing system (ECSMS) vs. calibrated secondary device.

As seen in Table 4, a low range and low SD throughout the 26 tests indicated that the system was consistent and corresponded to the accuracy of the secondary device during the repeatability tests. Furthermore, the low SD of each minor test indicated that the values were very close to the mean, and, in this case, little differences existed. To clarify, 26 segmented tests were evaluated, together generating over 1042 values. The average and variance values between the devices were close to perfect. It follows that the overall standard error of the mean of the differences and the SD of the mean of the major group were low (MD = −0.006°; SD = 0.045°). Likewise, a *p*-value of 0.997 signified that the two groups were near identical. There was no significant difference between the ECSMS and the secondary device during the repeatability test. In fact, both sets of data moved in a faultless stepped function to 125°, as presented in Figure 5. Note that the ECSMS only recorded when each angle was encountered (not in the zero/home position). There was a positive correlation (r = 0.999), thus indicating a perfect positive relationship during the test.

**Figure 6:**
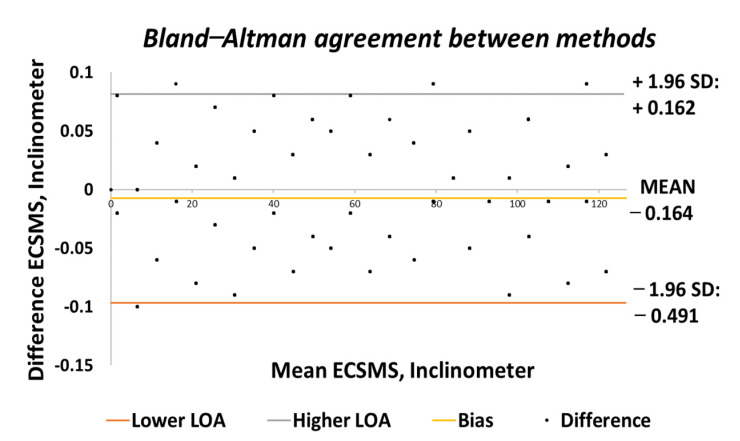
Repeatability test—agreement between the ECSMS and secondary device.

**Figure 7:**
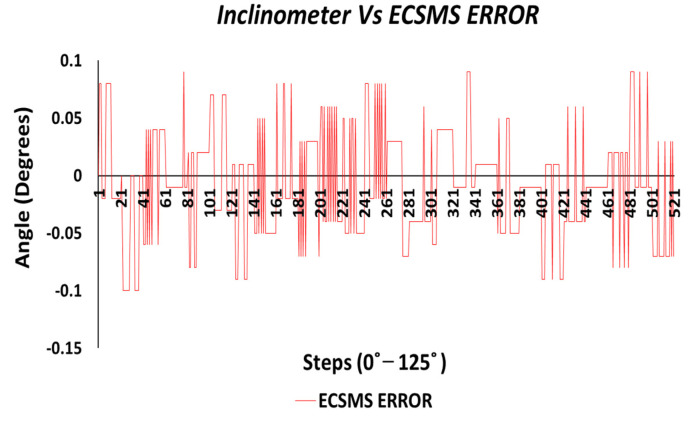
Repeatability test—ECSMS error vs. secondary device.

In conclusion, the two devices had an exceptionally good agreement and corresponded with the findings of the accuracy tests. The Bland–Altman chart presented in Figure 6 shows few outliers just outside the upper limit by a very small margin. However, these outliers were negligible when considering the vast amount of data (521 points) that overlapped (not visible due to high accuracy) within the upper and lower limits. Like the accuracy test, the low variability resulted in a narrow CI with a small margin of error (approximate lower LOA = −0.09° and upper LOA = 0.09°). In addition, the two devices showed an excellent correlation and were highly repetitive, and the scatter around the bias line was steady whilst also being variably consistent across the graph. Figure 7 displays the minimal error rate or the differences between the devices throughout the test.

To summarise, the systematic tests achieved the 0.1° accuracy of the secondary device, thus confirming that the system met the standards of the inclinometer. In addition, the findings showed a high agreement between devices and demonstrated that the ECSMS could deliver the same resolution, repeatability measures, and accuracy of the secondary device. It is important to recognise that the ECSMS was as accurate as the encoder (0.0087°), which was measuring the hinging arm itself. However, the ECSMS had essentially been downgraded because a secondary device could not be sourced with such accuracy to validate the system. Therefore, the statistical tests proposed the same accuracy of the secondary device regardless of the ECSMS encoder’s high specifications.

## 5. Smart Glove/Sensor Accuracy Testing

In the previous section, the results confirmed that the ECSMS device had a high accuracy and repeatability needed for a sensor measurement/monitoring system. Furthermore, the validated device brings support for future sensors/data gloves concerning evaluation and validation. This section provides a highly detailed evaluation of the accuracy of flex bend sensors, fibre optic sensors, IMU devices, and Hall effect technologies that have been previously researched for data gloves. Moreover, the sensor testing procedure followed the protocol shown in Section 4.1 (group 2a).

### Accuracy Testing of Different Sensor Technologies

The ECSMS and multiple sensors were stepped throughout the system’s range to follow the ROM of a finger joint. The Flexpoint sensor and the 5DT sensor were positioned into their unique capsules, as were the Hall effect and the IMU sensors. Each test was performed individually and closely followed the validation process of the ECSMS.

Note: the count amongst the technologies varied for the accuracy test. To clarify, the only reason these groups varied in the data range was because of the sampling rates and delays within the individual accuracy test programs that were uploaded at different times. In addition, the count between each technology and the ECSMS were identical and were all created to be significantly large in order to entail as much detail as possible between 0° and 125°, e.g., during the Flexpoint test, increments of 125°/1549 steps = 0.080° were performed and during the fibre optic test, increments of 125°/1283 steps = 0.097° were performed). Furthermore, each individual group test had separate descriptive and statistical methods performed like the ECSMS validation process.

**Figure 8:**
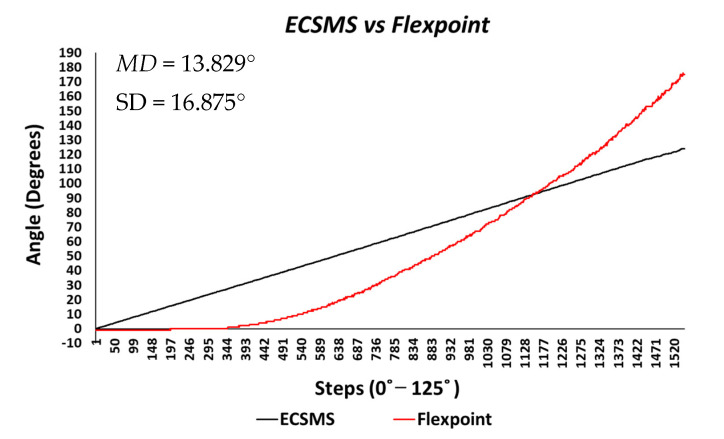
Accuracy test—ActionSense/Flexpoint bend sensor vs. ECSMS.

During the Flexpoint (ActionSense) test, the sensor’s output provided a non-linear response and had a low sensitivity, particularly in the low range. Though, in the higher range, the sensor was much more active and had an increased sensitivity. As a result, an increasing derivative response is presented in Figure 8.

The standard error of the mean of the differences and the SD of the mean of the differences were high (MD = 13.829°; SD = 16.875°). Similarly, the average and variance values between the two devices were dissimilar, as seen in Table 5. A *p*-value of $6.83\times 10^{-9}$ signified that the two devices had a poor relationship, and we rejected the null hypothesis $H_{O}:\mu_{1}=\mu_{2}=\mu_{k}$.

**Figure 9:**
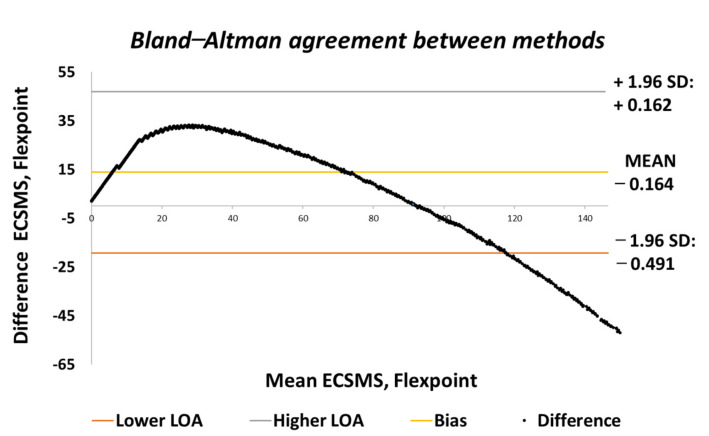
Accuracy test—agreement between Flexpoint bend sensor and ECSMS.

There was a significant difference between the ECSMS and the Flexpoint sensor. Nevertheless, there was a positive correlation (r = 0.956) that represented a good relationship, although the Bland–Altman chart in Figure 9 clarifies that there was no agreement between devices.

As can be seen, the trend was not repetitive and the scatter around the mean bias line was unsteady and variably inconsistent across the graph. For this reason, a high variability CI with a large margin of error (approximate lower LOA = −22° and upper LOA = 47°) was seen. It can be concluded that in this case, there was an unacceptable correlation, and the trend showed a case of proportional error—a case of absolute systematic error. Furthermore, the results provided an explanation to why the Pearson’s correlation value (r = 0.956) was much higher than expected. Hence, the variation in the lower range depended strongly on the magnitude of the higher range. Note: after 90°, the sensor showed a major derivative change. For instance, if the data’s maximum range was only selected to 90°, this would result in narrower CI with a reduced margin of error and a Pearson’s correlation value that would illustrate the poor relationship between devices.

**Figure 10:**
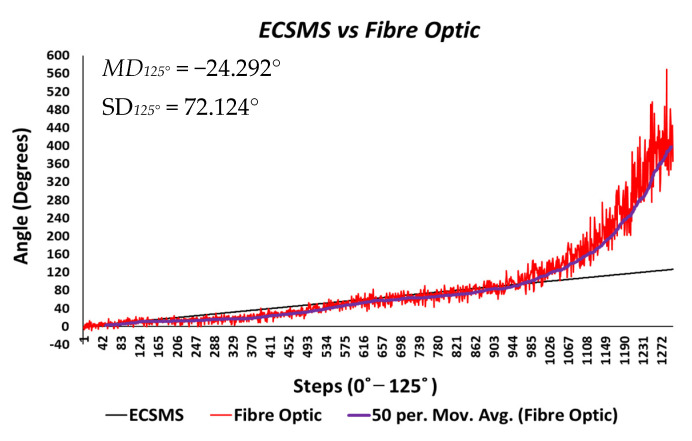
Accuracy test—5DT/fibre optic bend sensor vs. ECSMS with fibre optic moving average.

Throughout the fibre optic (5DT) sensor test, the sensor’s output produced a non-linear spiky response, as presented in Figure 10. For this reason, the standard error of the mean of the differences and the SD of the mean of the differences were very high (MD = −24.292°; SD = 72.124°). This response was not as deprived as the Flexpoint sensor in the low range but had the same non-linear reaction to angular rotation, especially between 20° and 60°. Furthermore, the sensor was much more sensitive to change, thus causing spikes throughout. However, the 5DT glove used filters to improve the dynamic range and accuracy, thus bringing the output closer to the moving average line (note: this did not resolve the non-linear response issues).

**Figure 11:**
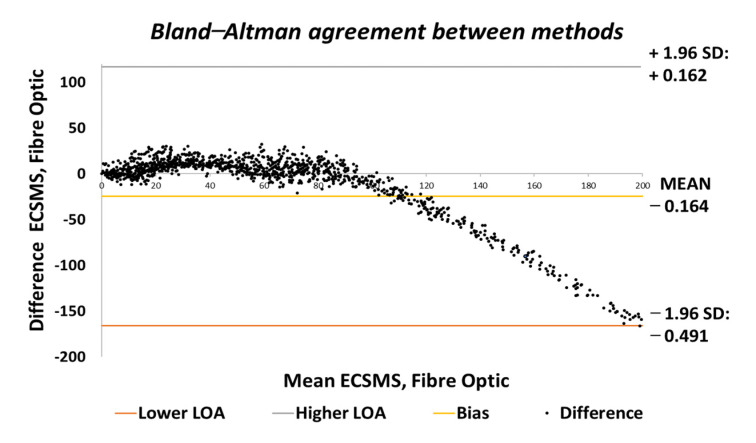
Accuracy test—agreement between fibre optic bend sensor and ECSMS.

Moreover, in the higher range, the sensor was much more active and had an increased error rate, as expected due to the optical fibre being strained considerably further at 90°. Like the Flexpoint sensor, after 90°, an increasing derivative response was present. The average and variance values between the two groups can also be seen to have been unrelated in Table 5. A *p*-value of $4.64\times 10^{-14}$ and a Pearson’s correlation value (r = 0.826) signifies that the two groups had a poor relationship, and we reject the null hypothesis. Due to the sensitive spiking nature of the data, the sensor’s output disadvantaged the overall *p*-value. Consequently, the Bland–Altman chart in Figure 11 shows a case of systematic error after 90°.

**Figure 12:**
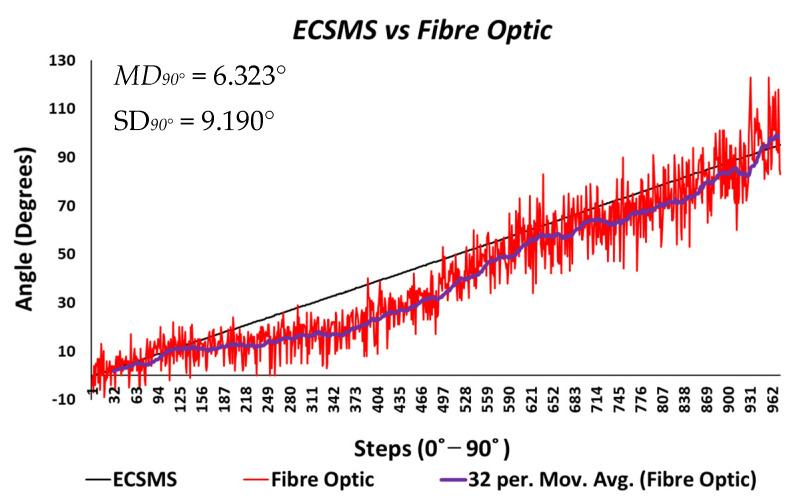
Accuracy test at 90°—fibre optic bend sensor vs. ECSMS.

The output showed a major derivative change after 90° to a higher extent than the Flexpoint sensor. In fact, the higher variability after 90° resulted in a wider CI with a large margin of error (approximate lower LOA = −160° and upper LOA = 110°). Additional sampling presented in Figure 12 demonstrates the values up to 90°, showing an improved correlation that indicated a good relationship.

**Figure 13:**
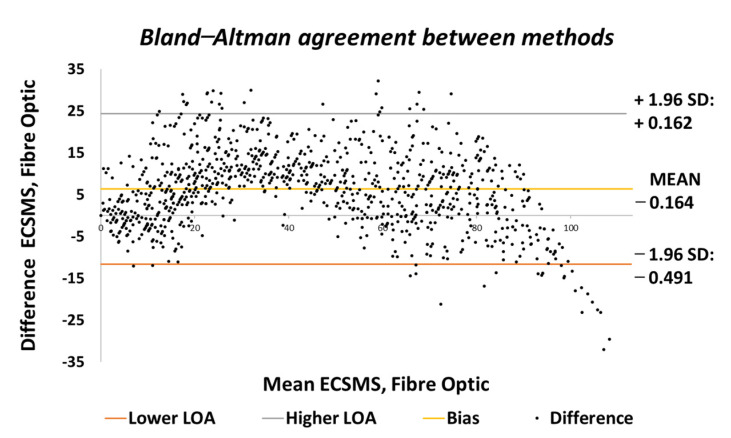
Accuracy test at 90°—agreement between fibre optic bend sensor and ECSMS.

A new *p*-value of $1.12\times 10^{-6}\;$ indicated the two series of data had a better relationship between 0° and 90°. For this reason, there is no doubt that if the fibre optic’s data were filtered, a much higher *p*-value or relationship would exist, thus matching the moving average line. Additionally, as the optical fibre was not strained past 90°, the derivative response in the high range was eliminated. Hence, the standard error of the mean of the differences and the SD of the mean of the differences were now much lower (MD = 6.323°; SD = 9.190°). Given the above, a positive correlation (r = 0.950) indicated a better relationship between the groups. The Bland–Altman chart (Figure 13) demonstrated a much better level of agreement between devices when the grouped data were only evaluated to 90°. The lower variability resulted in a narrower CI with a smaller margin of error (approximate lower LOA = −12° and upper LOA = 25°). Similarly, the scatter around the mean bias line was steady and variably consistent between 0° and 10° and between 65° and 90° but was inconsistent in the range of 20–65°.

**Figure 14:**
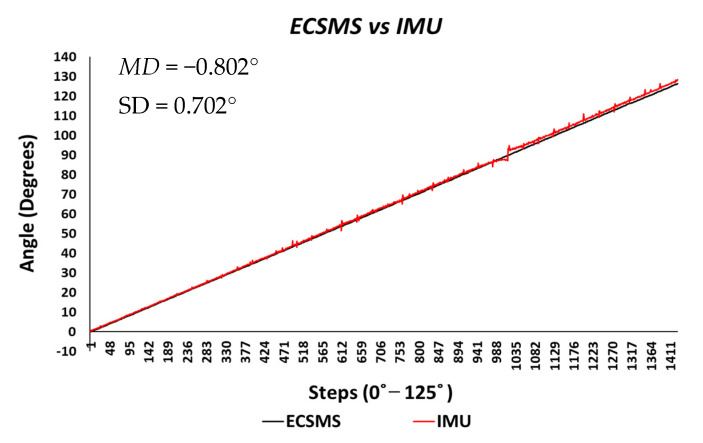
Accuracy test—IMU sensor vs. ECSMS.

Throughout the IMU test, the sensor’s output provided a brilliant linear response, as presented in Figure 14. The standard error of the mean of the differences and the SD of the mean of the differences were small (MD = −0.802°; SD = 0.702°). Furthermore, the average and variance values between the two devices were comparable, as seen in Table 5. A *p*-value of 0.560 implied that the two groups had a good relationship, and we accept the null hypothesis $H_{O}:\mu_{1}=\mu_{2}=\mu_{k}$. This signified that there was no significant difference between the ECSMS and the IMU sensor. Similarly, there was a positive correlation (r = 0.999), thus indicating an excellent relationship between the two groups.

**Figure 15:**
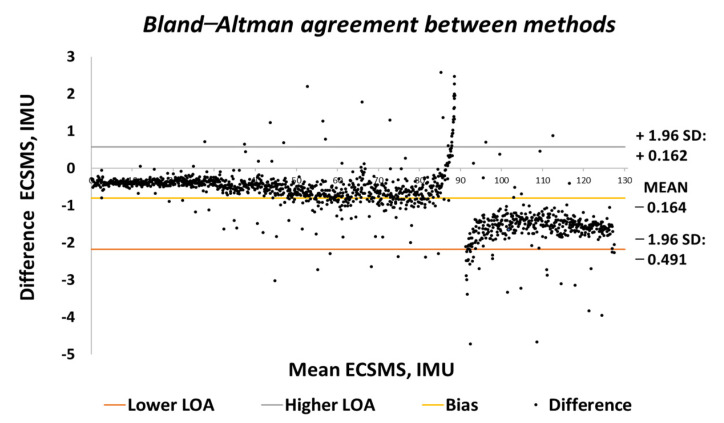
Accuracy test—agreement between IMU sensor and ECSMS.

The Bland–Altman chart in Figure 15 proves that there was a high level of agreement, where the trend was highly repetitive and the scatter around the mean bias line was steady and variably consistent up to 90°. However, at this point, a gimbal lock error is seen at the output.

In addition, a gimbal lock existed despite the upgrade in technologies because Euler angles (pitch, yaw, and roll) were not a mathematically complete representation of an object’s orientation. Fundamentally, at certain points, the degrees of freedom of the Euler angles could drop below 3. The problem could be efficiently eliminated by using a four-degree rotation freedom system. Furthermore, this is the mathematical equivalent of adding a fourth gimbal to the gyro. Moreover, quaternions are the most common alternative these days, since they have other properties that make them useful for calculations [22]. Nevertheless, after 90°, the device corrected itself, and the data followed on a steady and consistent path to 125°. Multiple outliers were seen to exceed the lower and upper LOA, which were the cause of the gimbal lock a 90°. Regardless, a low variability resulted in a narrow CI with a small margin of error (approximate lower LOA = −2° and upper LOA = 0.7°).

**Figure 16:**
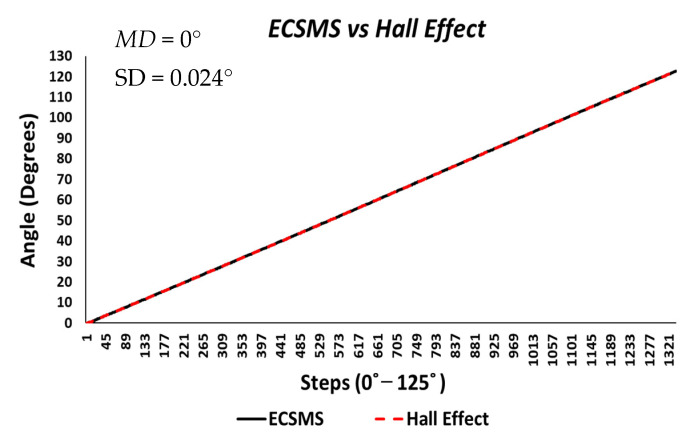
Accuracy test—Hall effect sensor vs. ECSMS.

Finally, the last accuracy test included a Hall effect technology. The ECSMS equipped with the sensor was stepped to 125° (as seen in Figure 16). The standard error of the mean of the differences and the SD of the mean of the differences were close to zero or non-existent (MD = 0°; SD = 0.024°). As a result, the sensor provided a flawless linear response that closely followed the ECSMS. Similarly, a Pearson’s correlation value of 1 indicated an identical relationship between the two sets of values.

**Figure 17:**
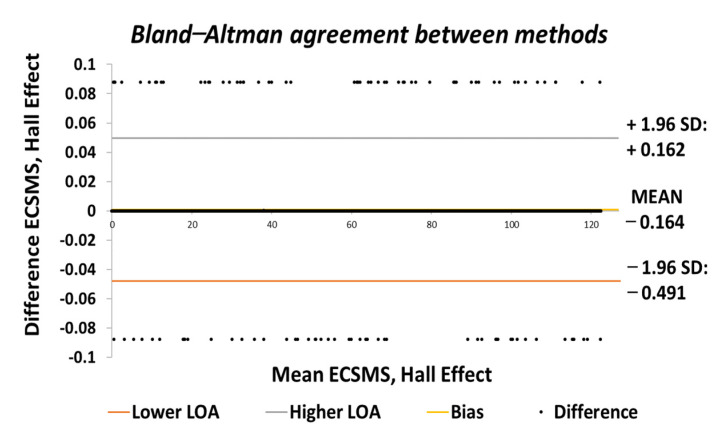
Accuracy test—agreement between Hall effect sensor and ECSMS.

Furthermore, the average and variance values between the two devices were near identical, as seen in Table 5. A *p*-value of 0.999 implied that the two groups had a near perfect relationship, and we accept the null hypothesis $H_{O}:\mu_{1}=\mu_{2}=\mu_{k}$. Hence, there were no significant differences between the ECSMS and the Hall effect sensor. Moreover, the concluding Bland–Altman chart in Figure 17 confirms that there was an astonishing level of agreement. In addition to this, the pattern was incredibly repetitive, and the scatter around the mean bias line was steady and variably stable up to 125°. However, several outliers were seen to exceed the lower and upper LOA but were well within the 0.1° range. The low variability resulted in a narrow CI with a small margin of error (approximate lower LOA = −0.05° and upper LOA = 0.05°).

**Figure 18:**
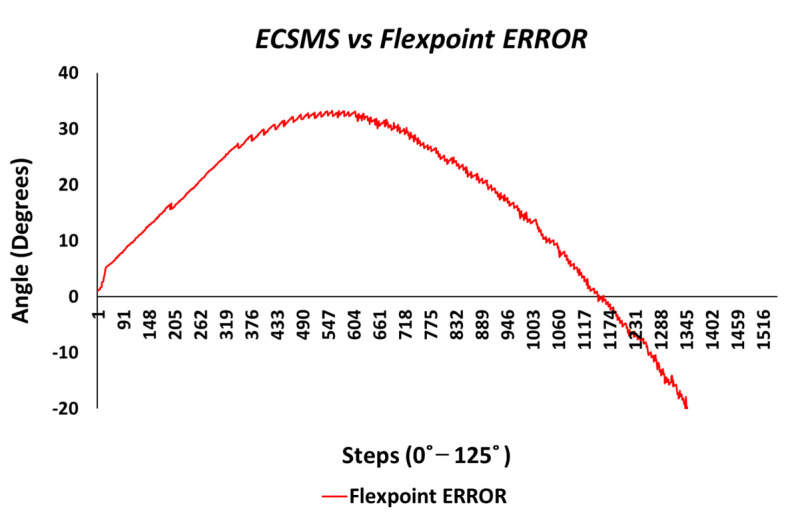
Accuracy test—Flexpoint bend sensor error vs. ECSMS.

The final part of the accuracy tests explored the errors/differences between the ECSMS and the sensor technologies. In this case, the differences between each group were plotted to demonstrate the magnitude of the errors across the full range of testing. Figure 18 reveals an inverted type illustration of the magnitude of the Flexpoint error. Without a doubt, the error was inconsistent and was maximum at mid-range bending (33°). As can be seen, the error was minimal at 90° when the sensor was in an active state. However, it was also seen that the error was minimal in the low range. Keeping the results seen in Figure 8 in mind, the lack of sensor inactivity or lack of response between 0° and 30° was in fact the cause of minimal error in the low range seen in Figure 18.

**Figure 19:**
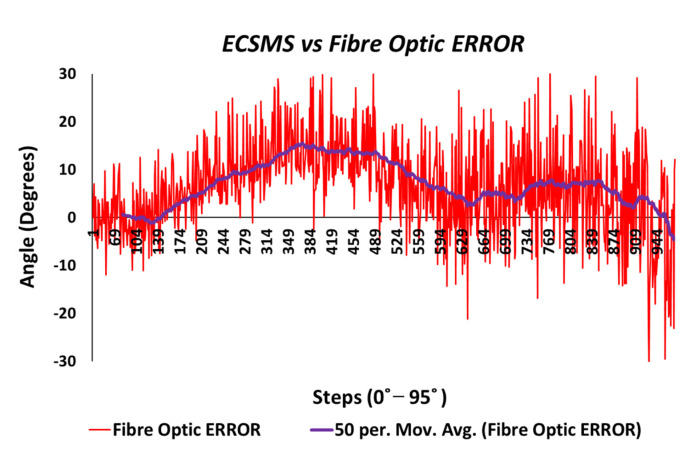
Accuracy test—fibre optic sensor error vs. ECSMS.

Figure 19 shows the magnitude of the fibre optic sensor error, where the error was spiky but consistent in certain ranges to 90°. However, in between 222 and 504 steps, the error peaked at 15° (in view of the moving average line/filter). As a result, the error surpassed 300° (graph scaled down for illustration purposes) once the optical sensor was under increased strain between 90° and 125°.

**Figure 20:**
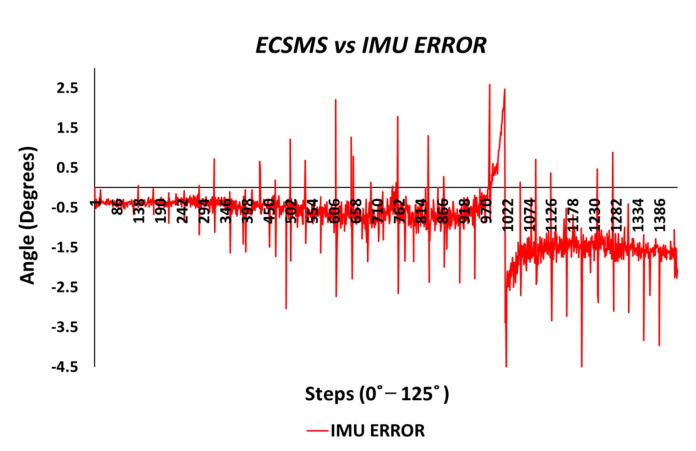
Accuracy test—IMU sensor error vs. ECSMS.

A gimbal lock error produced from the IMU is seen in Figure 20. If we disregard the gimbal lock, the magnitude of the error would be significantly low and consistent throughout the range (note: if quaternions are used to represent the rotations, no gimbal lock would be present in the device).

**Figure 21:**
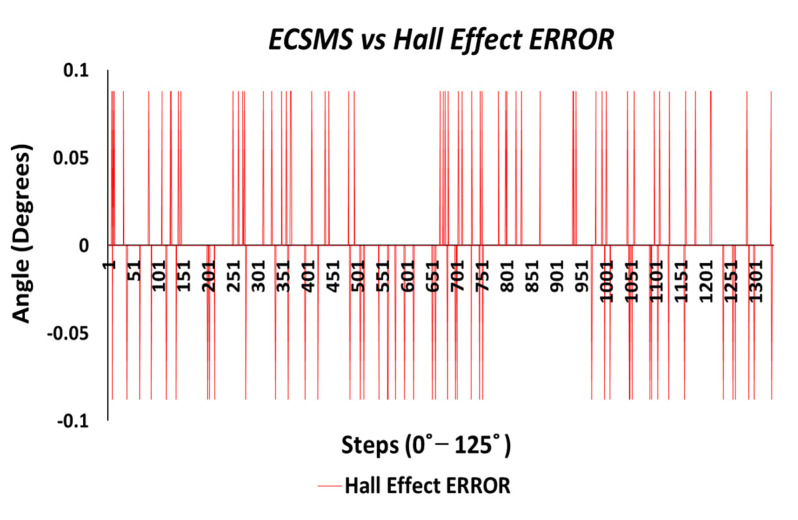
Accuracy test—Hall effect sensor error vs. ECSMS.

The Hall effect error was maximum at 0.09° and was extremely consistent across the range displayed in Figure 21.

## 6. Smart Glove/Sensor Repeatability Testing

In the previous section, the results provided a highly detailed confirmation that the accuracy levels of the flex bend sensors, fibre optic sensors, IMU devices, and Hall effect technologies that have been previously researched for data gloves. This section evaluates the repeatability measures of these sensors to follow the protocol shown in Section 4.1 (group 2b).

### Repeatability Testing of Different Sensor Technologies

Using the same statistical methods, this section compares the repeatability results of the sensors to evaluate the results under a certain set of procedures set in the protocol. Furthermore, the performance of these tests examined the sensor’s capability to consistently replicate angular readings against the ECSMS when implementing a dynamic testing strategy. A summary of the test results extracted from the ECSMS and each sensor is presented in Table 6. Moreover, the count for the repeatability steps was 460, and the overall outputted values were 920 within one group.

Just like the ECSMS’s validation process, one repeatability test contained multiple (22) segmented smaller tests (samples n = 20), each at approximately 5° apart. Equally, these tests were performed under the same conditions as the ECSMS validation process. Furthermore, the overall *p*-values and the Pearson’s coefficient values showed the relationship the sensors had with the validated system. Similarly, the trend followed the accuracy test findings of the last section (e.g., the IMU and the Hall effect outperformed the fibre optic and the flex bend sensor as expected).

**Figure 22:**
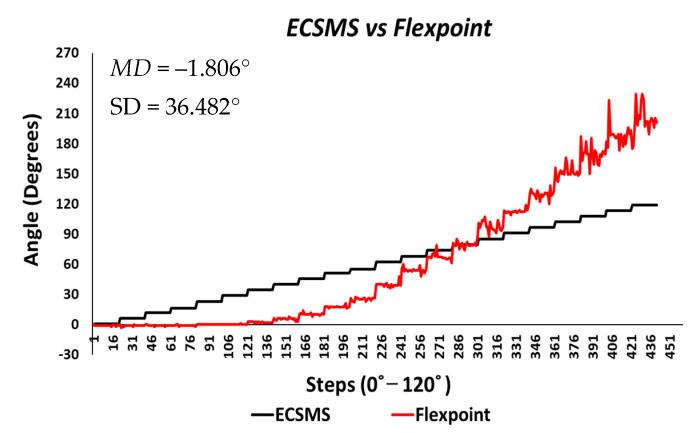
Repeatability test—Flexpoint bend sensor vs. ECSMS.

The Flexpoint repeatability test displayed interesting characteristics of the sensor. Keeping the poor results from the accuracy test in mind, the sensor unexpectedly followed the ECSMS’s linear step function to a small degree, as seen in Figure 22. The total standard error of the mean of the differences and the SD of the mean of the differences were MD = −1.806° and SD = 36.482°. The MD was relatively low when considering the sensor output illustrated. In the low range, the sensor had a poor performance and in the high range, and an increasing derivative change equivalent to the accuracy test was seen. In this case, a variation in the low range strongly depended on the magnitude of the high range. Hence, giving a high *p*-value of 0.708 and a Pearson correlation (r = 0.937) (seen in Table 6). Surprisingly, the sensor performed better at mid-way bending during repeatability measures (e.g., the steps tended to equal out with little spikes/changes), although it was clear that the accuracy was still not met.

Each segmented test seen in Table 7 provided 22 individual values for the standard error of the mean of the differences and the SD of the mean of the differences. It can be concluded that the flex bend sensor did not start operating as it should have until approximately 30°, disadvantaging the overall repeatability results. Furthermore, the mean values of each group test did not correspond to the true value of the ECSMS throughout the range. However, the mean of the values of groups between 70° and 90° was much closer as the sensor’s accuracy increased, as expected from earlier results.

**Figure 23:**
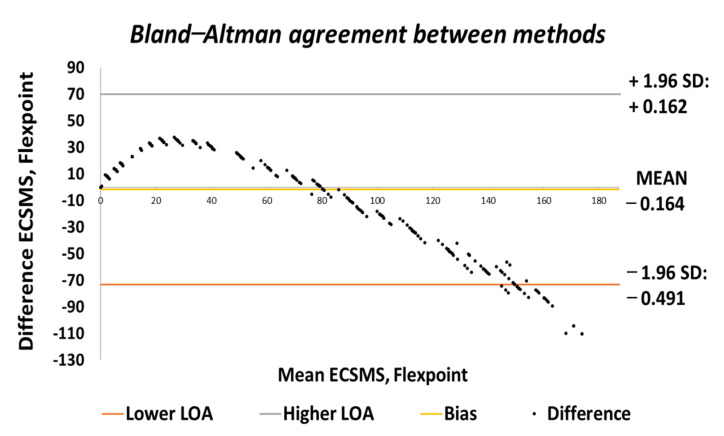
Repeatability test—agreement between Flexpoint bend sensor and ECSMS.

Moreover, the SD values deviated from the mean values in the higher range. Figure 23 shows similar characteristics on a Bland–Altman chart, where it can be seen the higher variability resulted in a wide CI with a large margin of error. In this case, there was an unacceptable correlation, and the trend showed a case of related error to the accuracy results.

**Figure 24:**
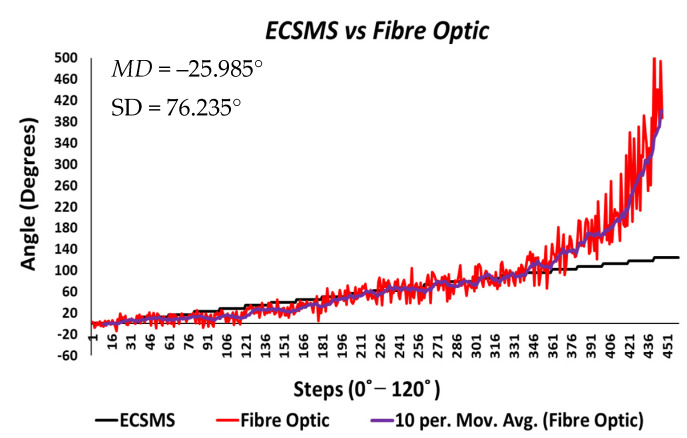
Repeatability test—fibre optic sensor vs. ECSMS with fibre optic moving average.

Figure 24 displays the fibre optic sensor’s characteristics throughout the full range to 120°. A low *p*-value of $4.8182\times 10^{-7}$ and a Pearson correlation (r = 0.812) is seen in Table 6. These results were also less than satisfactory during the 22 segmented tests. Moreover, the findings influenced a second representation of the repeatability test, though only to a maximum range of 90°.

**Figure 25:**
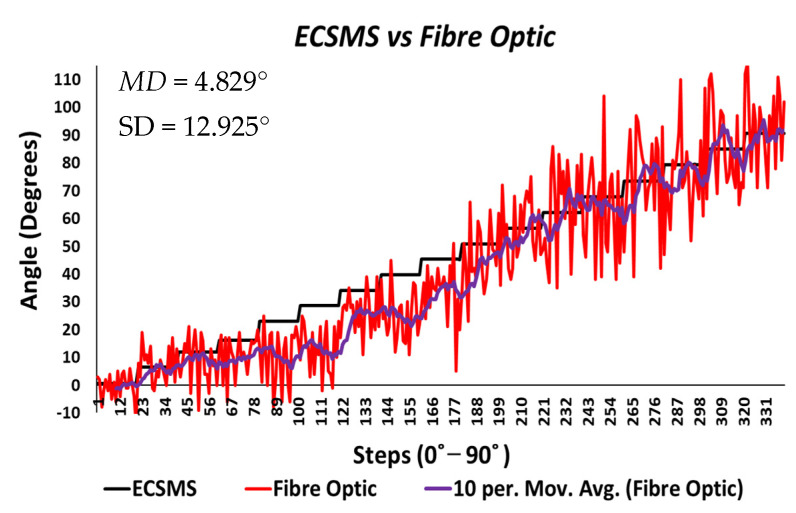
Repeatability test at 90°—5DT/Fibre optic bend sensor vs. ECSMS average.

The results seen in Figure 25 display a much stronger response to linearity than the Flexpoint sensor. The overall standard error of the mean of the differences and the SD of the mean of the differences are reduced from MD = −25.985° and SD = 76.235° to MD = 4.829° and SD = 12.925°. A higher *p*-value of 0.036 and a Pearson correlation (r = 0.917) is seen in Table 6. However, with the sensor filtered (modelled by the moving average line), the sensor repeatability test was not as convincing as the Flexpoint sensor (no visible steps to match the ECSMS).

**Figure 26:**
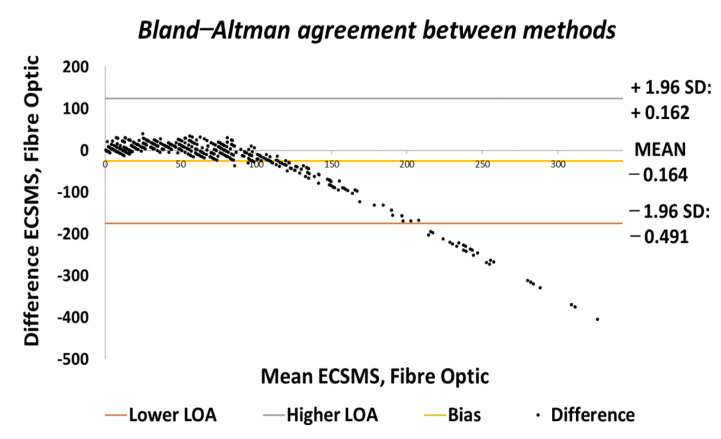
Repeatability test—agreement between fibre optic bend sensor and ECSMS.

**Figure 27:**
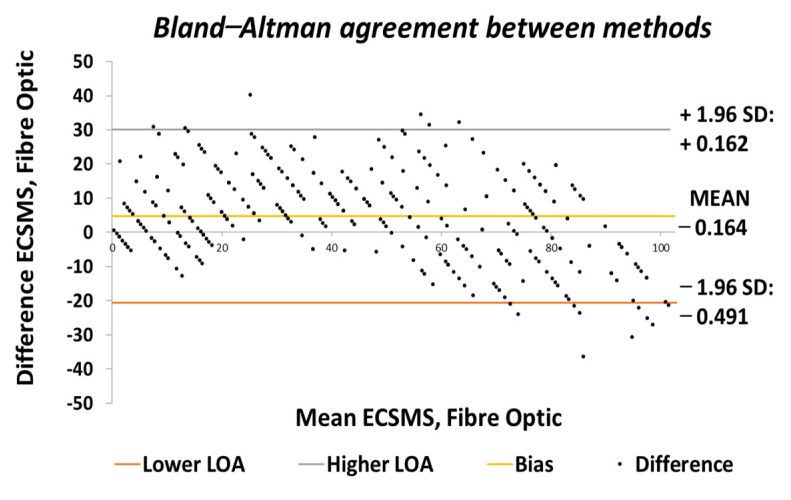
Repeatability test at 90°—agreement between fibre optic bend sensor and ECSMS.

Moreover, the Bland–Altman chart seen in Figure 26 demonstrates similar results to the sensor accuracy test in the last section with a wide CI and high error rate after 90°. Another Bland–Altman chart (Figure 27) proves a decreased error rate below 90° with a smaller CI, as expected from the accuracy results.

Table 8 displays 22 individual repeatability tests that provide separate values for range, MD, and SD. In addition, the range between each group was significantly large due to the spiky nature of the data but increased exponentially near 90°. Nevertheless, the mean values for each repetitive group were respectable (all within 3°) up to 90° except for the affected area between 10° and 45°.

To clarify, as mentioned during the accuracy test, the 5DT glove uses smoothing filters within its software to overcome the spiky nature of the output. Consequently, the spiky nature in the optical signal occurs when the transmitted light is affected by impurities while bending [23]. However, smoothing filters do not fix the non-linear characteristics seen in Figure 25 (seen by the moving average line) and the dramatic derivative change after 90° (seen in Figure 24).

**Figure 28:**
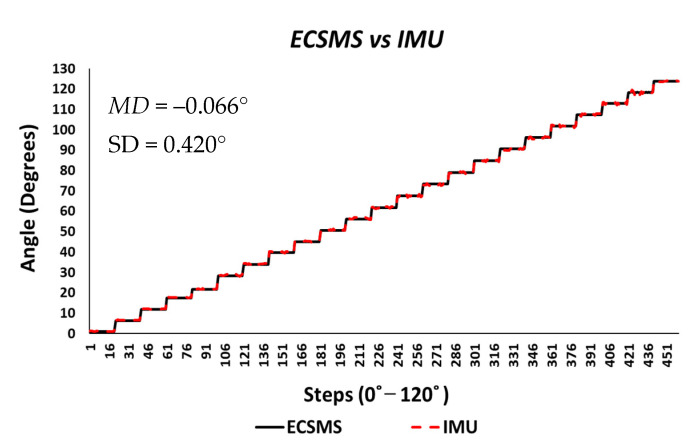
Repeatability test—IMU sensor error vs. ECSMS.

The vigorous repeatability tests performed on the IMU provided excellent results. To illustrate, the results seen in Figure 28 verified the strong relationship between the ECSMS and the sensor (as seen in Table 6 and Table 9).

**Figure 29:**
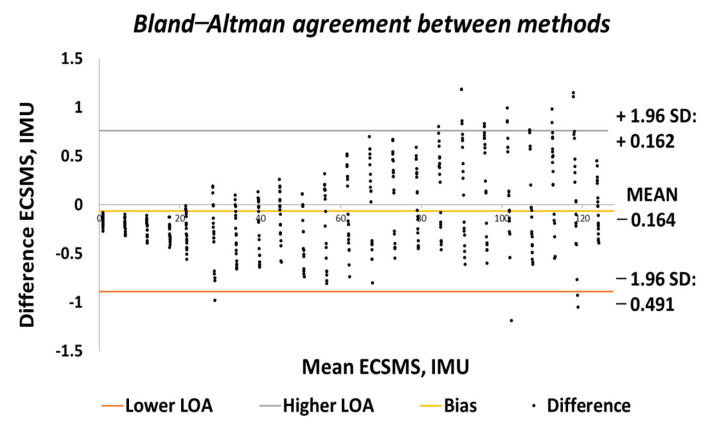
Repeatability test—agreement between IMU sensor and ECSMS.

Both sets of data moved in the same direction in a stepped function that denoted the 22 tests. A *p*-value of 0.978 (seen in Table 6), implied that the two groups had an excellent relationship, and we accept the null hypothesis $H_{O}:\mu_{1}=\mu_{2}=\mu_{k}$. Likewise, this signified that there was no significant difference between the ECSMS and the IMU sensor during the repeatability tests. Similarly, there was a positive correlation (r = 0.999), thus indicating an excellent relationship between the two devices. The overall standard error of the mean of the differences and the SD of the mean of the differences were low (MD = −0.066°; SD = 0.420°). For this reason, the Bland–Altman chart seen in Figure 29 shows a narrow CI with a small margin of error (approximate lower LOA = −0.09° and upper LOA = 0.09°).

Furthermore, each of the segmented test seen in Table 9 provided 22 individual values for the standard error of the mean of the differences and the SD of the mean of the differences. A small increase in range towards 90° had a minor effect on the SD, as the mean of the differences were slightly increased. Note: Euler angles were only used to evaluate the IMU characteristics against the ECSMS for this experiment. Undertaking this, a gimbal lock was present, as discussed in Section 5. Hence, if quaternions were used to represent the rotations, no gimbal lock would have been displayed on the device.

**Figure 30:**
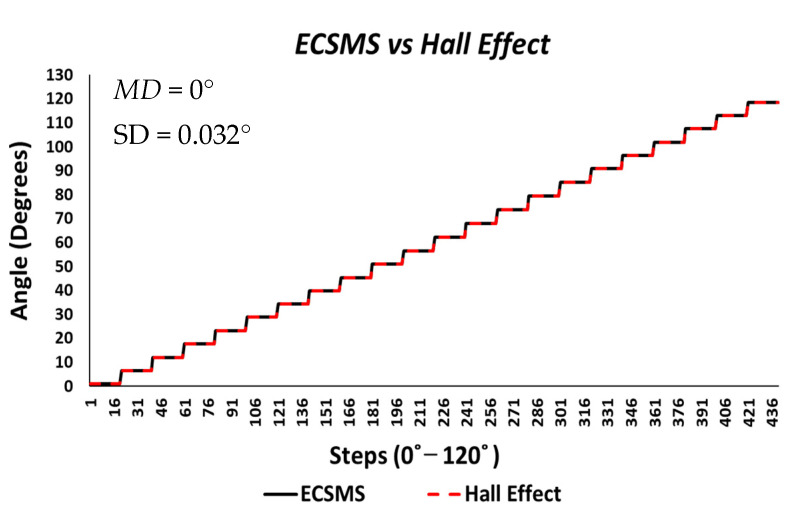
Repeatability test—Hall effect sensor error vs. ECSMS.

**Figure 31:**
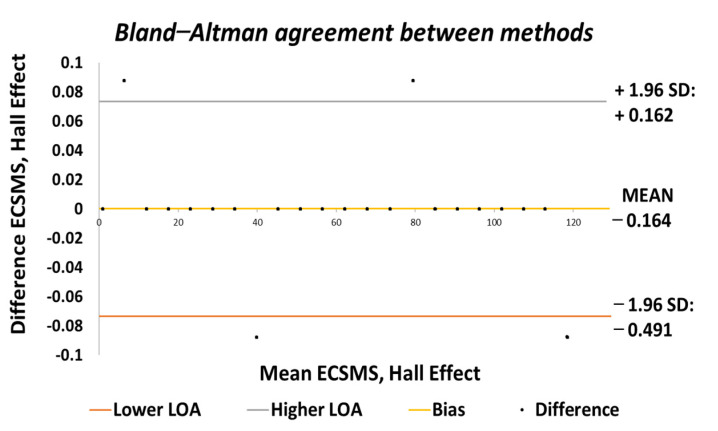
Repeatability test—agreement between Hall effect sensor and ECSMS.

Finally, the Hall effect sensor was tested to determine its capability to uphold consistently replicated angular readings of the ECSMS. For the fourth and final time, the ECSMS was stepped 460 times to approximately 120°. A stronger relationship is seen in between the ECSMS and the sensor (seen in Figure 30), where the overall standard error of the mean of the differences and the SD of the mean of the differences can be seen to have been zero or non-existent (MD = 0°; SD = 0.032°). Furthermore, the Bland–Altman chart seen in Figure 31 shows in a narrow CI with an extremely small margin of error (approximate lower LOA = −0.07° and upper LOA = 0.07°). A small number of outliers rested on the outside of the upper and lower limits. However, these were acceptable as only few existed and were within proximity of both the upper and lower LOA.

During each of the minor tests (as seen in Table 10), the minimum and maximum values were similar if not identical. As a result, this reduced the overall range between the devices, and the standard error of the mean of the differences and the SD of the mean of the differences were near to zero or true zero. Furthermore, these results were similar to the IMU sensor, as both sets of data moved in the same direction in a stepped function that denoted the 22 tests. Moreover, a *p*-value of 0.999 (as seen in Table 6), implied that the two groups had an excellent relationship, and we again accept the null hypothesis $H_{O}:\mu_{1}=\mu_{2}=\mu_{k}$. Consequently, this suggests that there were no significant differences between the ECSMS and the Hall effect sensor. Likewise, there was a positive correlation (r = 0.999), thus indicating an excellent relationship between the two devices.

The final part of this thorough study explored the errors/differences between the ECSMS and the sensor technologies during repeatability measures. To demonstrate the magnitude of the errors across the full range of testing, the differences between each group were plotted.

**Figure 32:**
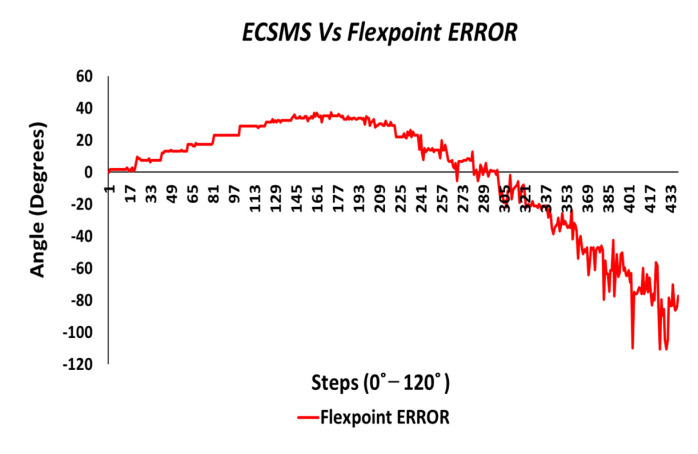
Repeatability test—Flexpoint bend sensor error vs. ECSMS.

Figure 32 shows an inverted type illustration of the results found of the repeatability test in Figure 22. This error was inconsistent across the range and could be read from two different perspectives: repeatability and accuracy.

Additionally, the repeatability error was best in the mid-range because the steps were not visible. In addition, where the steps were clearly visible, the repeatability error was higher. As reported in the accuracy section, the Flexpoint sensor underperformed in the low range, which added to the factors during the repeatability test. Moreover, the non-linear response (positive error) was maximum approximately 40°, and the error between the devices was seen to reduce at 90°. At this point, the sensor output increased exponentially in a positive direction. The negative error seen in the chart was large at −120°.

**Figure 33:**
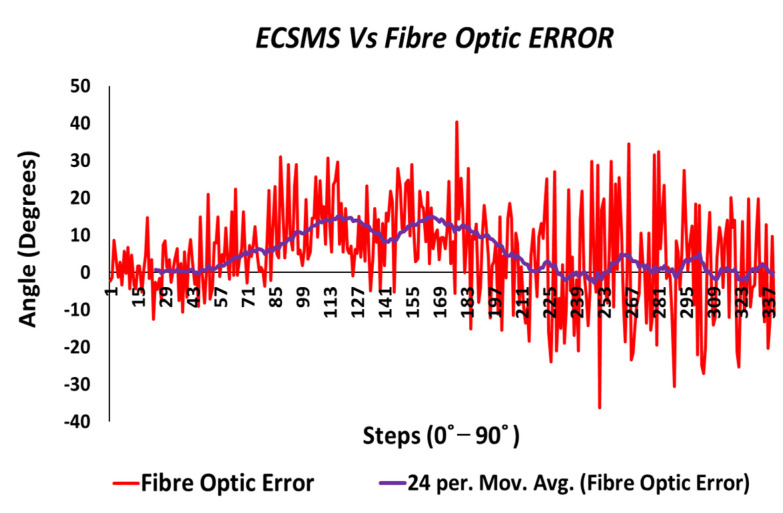
Repeatability test at 90°—Hall effect sensor error vs. ECSMS.

For illustration purposes, the fibre optic error was only evaluated to 90° for the clarity of the sensor’s normal working range. As a result, the sensor error was much more consistent (as seen in Figure 33) but was hugely affected by impurities, while bending causing a spiky output.

Furthermore, a moving average line was added to mimic how the data would look if filtered by the controlling software. With respect to the actual data, a maximum positive error of only 14° was visible near mid-range bending. Moreover, the sensor’s error was good from 225 to 337 steps (65–90°), with a maximum of approximately 6° and a negative error of approximately 3°.

**Figure 34:**
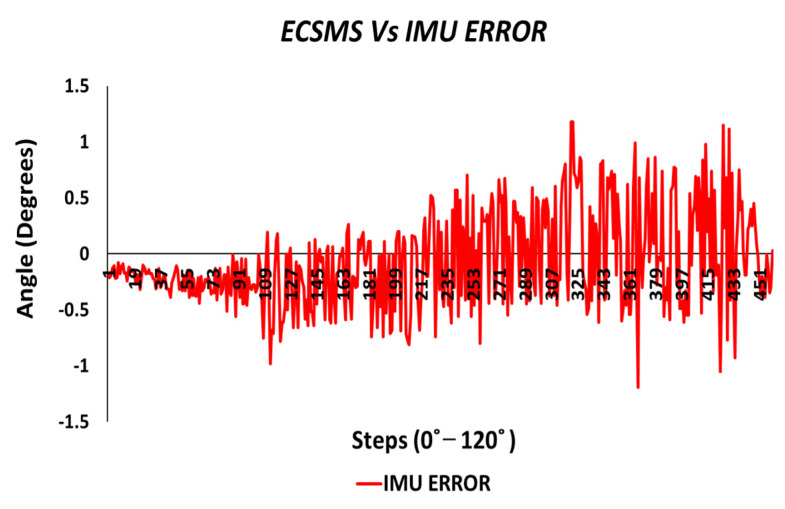
Repeatability test—IMU sensor error vs. ECSMS with IMU moving average.

The error between the IMU and the ECSMS (as seen in Figure 34) was minimal at a maximum range of approximately 1°. As discussed previously, the gimbal lock added to the error between the devices and can accordingly be ignored.

**Figure 35:**
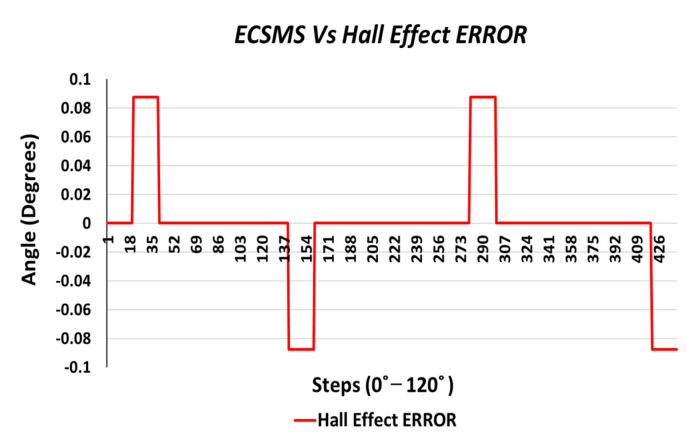
Accuracy test—Hall effect sensor error vs. ECSMS.

Finally, the last test evaluated the error between the ECSMS and the Hall effect sensor (as seen in Figure 35). The sensor had a positive maximum error of 0.09° and negative error of 0.09°, where the error only occurred within four points between 0 and 120°.

## 7. Discussion

This study proposed a state-of-the-art measurement system for monitoring data glove technologies. The system was designed to have high precision and compactness, as well as to allow for the total functionality of the moving arm. In addition, the system was designed to mimic finger joint movement from hyper extension to full flexion.

All data gloves/sensors were verified under dynamic conditions because they must be able to record the parameters of a finger’s continuous motion in most applications. To verify the accuracy, reliability, and stability of the system, two verification tests were set and conducted. First, the ECSMS’s accuracy was verified against a recognised secondary device. The descriptive statistics showed a high correlation between the device and the ECSMS, where both the resolution and accuracy were consistent throughout the full range. Secondly, the ECSMS’s repeatability was verified against the same secondary device, as it appeared to uphold the 0.1° accuracy and resolution during the initial test. Furthermore, the results showed a high correlation as the ECSMS outputted an identical stepped function through all the 26 minor tests. Moreover, a null hypothesis, which stated that there is no difference between the two groups, was formulated. By the end, the null hypothesis was not rejected because the *p*-value was close to 1 for both groups of data (accuracy and repeatability).

The second part of the study verified the accuracy, reliability, and linearity of different sensor technologies that are currently being used in data gloves. These results are revolutionary in the area of data gloves because clear characteristics were seen of the four technologies.

The results of the Flexpoint sensor showed a bad correlation, as a nonlinear response was recognised throughout the whole range. The *p*-value and the Pearson’s correlation value were good. However, Bland–Altman charts showed that the variation in the lower range depended strongly on the magnitude of the higher range. Furthermore, the sensor did not perform as it should have in the low range and only started outputting data at approximately 30°. Moreover, at mid-range bending during the repeatability test, the sensor showed signs of good repetitive measures. However, accuracy was not met until approximately 90°. At this point, the repeatability measures reduced, and the sensor output grew exponentially in a positive direction.

During the fibre optic sensor test, the results were initially discouraging, but further investigation using technical analysis tools filtered the data and showed good results. Because of the spikiness/attenuation of light, the *p*-value and correlation values were bad. As a result, the reduced light attenuation was proven to have a huge effect after 90°. Additional Bland–Altman charts showed large CI, but with the use of specific technical analysis tools to represent smooth data, a good linear response was seen throughout the range towards 90°. Furthermore, this response was slightly discouraged at mid-range but immediately ramped up to match the 90° range of the ECSMS. Moreover, the sensor response was much better in the low range and was much more linear than the Flexpoint sensor at mid-range bending. However, both sensors’ outputs increased exponentially after 90° due to strains on the optical fibre and added/changing variables (radius) to the flexible substrate.

The results for the IMU showed high correlation and relationship, as the *p*-value and Pearson’s coefficient values were near 1. Consequently, the SD and MD were very low, and the Bland–Altman charts demonstrated narrow CI lines throughout the whole range. Euler angles were used for the purpose of this study, where a gimbal lock was demonstrated at 90°. Furthermore, the error results showed less than 1° with few outliers that could have been the cause of the gimbal lock.

Finally, the Hall effect technology was the last sensor to be evaluated. The initial results showed a high agreement with the ECSMS, as the *p*-value and Pearson’s co-efficient were close to 1 during the accuracy and repeatability tests. This suggests that the sensor had the same relationship as the high-precision encoder of the ECSMS, with an accuracy of 0.1° with identical repeatability measures. Similarly, the MD and the SD were close to zero if not zero, meaning the Hall effect technology was found to lie within the same level of accuracy and repeatability as the ECSMS’s encoder. Furthermore, the Bland–Altman charts demonstrated the low variability results in a narrow CI with a slight margin of error.

To clarify, the ECSMS was validated with the same accuracy and resolution that met the 0.1° of the secondary device. However, its capability was much higher, as the device could be stepped 256 times inside 1 step (1.8°/256 = 0.007°), whilst the ECSMS’s encoder measured the smallest resolution/accuracy of 0.0087°.

## 8. Conclusions

Data gloves have been used in several applications, such as robotics and virtual reality, but researchers have turned their attention towards medical applications in recent years [24]. This paper primarily emphasises the importance of accurate hand functional assessment used to assess RA, PD, and other neurological conditions. Current RA detection methods rely on observation, questionnaires, and physical measurements, each of which has its own limitations. Clinicians and researchers have identified ROM as the key indicator of joint stiffness, and it is commonly assessed using a UG device to determine joints restriction. However, even when calculating static ROM, the UG is not without its drawbacks. It is perhaps no surprise then that the reliability of each small joint is reduced independently, given that the human hand consists of 27 bones of approximately 25 DOF. Additionally, morning stiffness is not calculated by rheumatologists because it can only be measured in the first 30 min of a person’s day.

Sensor gloves have the capability of remotely monitoring morning stiffness and of assessing hand function, although their realistic measures are still limited by accuracy, repeatability measures, weight, size, wear ability, and cost. The unknown physiological effects and reduced repeatability of sensor technologies have constrained the use of devices into the clinical practice. Consequently, wearable technology should be capable of continuously monitoring hands without causing obstruction to the daily tasks of the patient.

Previous researchers [1,3,15] have assessed data glove technologies, but the margin for error during sensor evaluation is substantial and only limited to static measurements. In addition, it is important that the collected data are trustworthy regarding wearable technologies to eliminate false data being accepted and good data being rejected. Equally, this adds to wasted time, wasted money, and the uncertainly of the technology’s characteristics throughout the research.

A roadmap for designing a state-of-the-art dynamic data/glove sensor measuring device to assess recent smart glove technologies is provided by this paper. To the best of our knowledge, this is the first application of a sensory glove/measurement system that closely resembles real finger joint movement (hyperextension and flexion–extension). Furthermore, the system can provide dynamic movements with a resolution of 0.007°, and its output can be validated against a recognised secondary device with 0.1° precision.

This paper addresses the main limitations of data gloves by precisely monitoring different sensor technologies. The results provide strong evidence regarding sensor technologies used to assess ROM. Such results suggest that clinicians can use a range of sensors depending on the device’s intended application. To summarise, the thorough results make it possible for researchers in the field of wearable sensors to overcome the limitations found in this paper.

As technologies advance, there are very exciting prospects for high-potential devices in hand rehabilitation and, in general, healthcare services. Consequently, there is a need for an ambulatory system capable of assessing regular improvements in patient activity to recognise the symptom of joint stiffness. Therefore, a practical and reliable device that can record several parameters of hand motion is required to assist with disease diagnosis and in the field of rehabilitation.

In conclusion, this research proposes further investigation into contact systems by carefully considering various technologies and how they can benefit hand functional assessment. Numerous data gloves have been proposed to date, but none have created a benchmark system that can monitor data glove/sensor technologies to such high level of precision. This work attempts to address the inaccuracy of data gloves by specifically studying each sensor using the ECSMS system.

## 9. Future Work

Current limitations of sensor monitoring systems are that no capacitive bend sensors are tested by the ECSMS. Therefore, future assessments on capacitive bend sensors will be completed. Additionally, the moving finger does not model the shape of a human finger for a glove attachment/fitment. As a result, the ECSMS has a modular design and allows the operator to interchange the arm or to adopt software to suit their needs in the future. Likewise, the hinging mechanism was designed to allow for an additional textured finger-like object to be attached to match a real human joint. In addition, this will benefit the field by adding sensors to dummy skin or by placing a glove on the device to be monitored to a validated high precision of 0.1°. The evidence and contributions specify that the proposed system has the potential to benefit smart glove applications in the practical rehabilitation field.

In the future, attempts will be made to produce a smart glove device using technologies that have been evaluated in this study. The ECSMS brings new possibilities to this area and reduces the need for early clinical experiments for hand function assessment to aid the design of a novel smart glove. Furthermore, the accuracy and repeatability of hand kinematics is essential and should also be authenticated prior to large-scale clinical trials in order to ensure a certain outcome for sensorised gloves. Moreover, power management, connectivity, signal processing, calibration, exoskeleton devices, and textile materials will also be included in future studies to develop an optimised and accurate automated hand function evaluation device.

## Figures and Tables

**Table 1:** ECSMS (list of components and test sensors).

Reference Number	Reference	Name	Description
**0**	**A**	Hall effect sensor	Hall effect test sensor
**1**	**B**	Stepper motor	Pivoting arm control
**2**	**C**	Micro switch	Homing circuit zero position
**3**	**D**	Fibre optic bend sensor	5DT (Fifth Dimension Technologies) glove test sensor
**4**	**E**	Flexpoint sensor	Flexpoint test sensor (ActionSense)
**5**	**F**	Fibre optic capsule	Fibre optic sensor polycarbonate positional holder
**6**	**G**	5 mm shaft	Main driven shaft
**7**	**H**	Flexpoint signal wires	Signal out/communication wires
**8**	**I**	Platform top	Rest point parallel to base
**9**	**J**	Fibre optic signal wires	Signal out/communication wires
**10**	**K**	ECSMS (high-precision encoder)	Validated sensor
**11**	**L**	Strong fixing bracket	ECSMS sensor bracket
**12**	**M**	ECSMS tower	Perpendicular tower
**13**	**N**	ECSMS adjustment	Adjustment for reference zero position
**14**	**O**	Switch B	(AC Board) Dynamic arm trigger
**15**	**P**	Switch A	(Z Board) Sensor circuit trigger
**16**	**Q**	Beeper/signal	Specified angle met/record point
**17**	**R**	Flexpoint circuit	Flexpoint voltage divider circuit
**18**	**S**	Stepper motor driver	Stepper motor driver carrier
**19**	**T**	Stepper motor Indicators	Homing, run and finished indicators
**20**	**U**	Capacitor	Decoupling capacitor
**21**	**V**	Custom adaptable sensor board	Sensor interchangeable link board
**22**	**W**	Fibre optic circuit	Photodiode signal/divider circuit
**23**	**X**	Sensor board reset	Reset the sensor board
**24**	**Y**	Sensor board power	Sensor board DC power jack
**25**	**Z**	Sensor board port	USB port (Arduino 1)
**26**	**AA**	Stepper board power	Stepper board DC power jack
**27**	**AB**	Polycarbonate holder	Holder for all components
**28**	**AC**	Stepper board port	USB port (Arduino 2)
**29**	**AD**	ECSMS base	Base with hidden rubber feet
**30**	**AE**	Main power stepper driver	Stepper motor main hold current/power
**31**	**AF**	Ferrite clamp	Split ferrite core/EMI (elecrtomagentic interference) reducer
**32**	**AG**	Inertia measurement unit (IMU) out (twisted pair)	Signal out/communication wires
**33**	**AH**	Bearing support	High-precision ball race bearings
**34**	**AI**	Pivoting arm	Aluminium controllable arm/finger model
**35**	**AJ**	IMU sensor (9 degrees of freedom (DOF))	IMU test sensor
**36**	**AK**	Polycarbonate surface/capsule	Cover to contain moving Flexpoint sensor
**37**	**AL**	Fibre optic guide	5DT fibre optic sensor polycarbonate guide
**38**	**AM**	Moving arm	Homing circuit lever/arm
**39**	**AN**	Switch lever	Homing circuit lever/trigger

**Table 2:** Accuracy statistical tests (ECSMS vs. secondary device).

Summary						
Groups	Count	Sum	Average	Variance	*p*-Value	Pearson’s Correlation (r)
ECSMS	1234	74,815.66	60.62857	1233.37	0.99536328	1
Inclinometer	1234	74,825.8	60.63679	1233.394		

**Table 3:** Repeatability statistical tests (ECSMS vs. secondary).

Summary						
Devices	Count	Sum	Average	Variance	*p*-Value	Pearson’s Correlation
ECSMS	521	32,134.6	61.67869	1313.351868	0.9977499	0.999999999
Inclinometer	521	32,137.9	61.68503	1313.632775		

**Table 4:** Repeatability test (ECSMS vs. secondary device).

ECSMS vs. Secondary Device						
Finger/Arm Angle and Method of Measurement	N	Min	Max	Mean	Range	SD
1.58	20	1.5	1.6	1.565	0.1	0.048
6.5	20	6.5	6.6	6.555	0.1	0.051
11.34	20	11.3	11.4	11.33	0.1	0.047
16.09	20	16	16.1	16.095	0.1	0.022
20.92	20	20.9	21	20.92	0.1	0.041
25.67	20	25.6	25.7	25.66	0.1	0.05
30.41	20	30.4	30.5	30.43	0.1	0.047
35.25	20	35.2	35.3	35.28	0.1	0.041
40.08	20	40	40.1	40.08	0.1	0.041
44.83	20	44.8	44.9	44.825	0.1	0.044
49.66	20	49.6	49.7	49.66	0.1	0.05
54.15	20	54.1	54.2	54.17	0.1	0.047
58.98	20	58.9	59	58.955	0.1	0.051
63.73	20	63.7	63.8	63.73	0.1	0.047
68.56	20	68.5	68.6	68.595	0.1	0.022
74.54	20	74.5	74.6	74.52	0.1	0.041
79.29	20	79.2	79.3	79.28	0.1	0.041
84.21	20	84.2	84.2	84.2	0	0
88.25	20	88.2	88.3	88.28	0.1	0.041
93.09	20	93.1	93.1	93.1	0	0
98.01	20	98	98.1	98.04	0.1	0.05
102.76	20	102.7	102.8	102.785	0.1	0.036
107.59	20	107.6	107.6	107.6	0	0
112.42	20	112.4	112.5	112.42	0.1	0.041
116.99	20	116.9	117	116.97	0.1	0.047
121.83	20	121.8	121.9	121.88	0.1	0.041
N = total number of measurements						
SD = standard deviation						

**Table 5:** ECSMS vs. glove technologies accuracy test results.

Summary							
Groups	Devices	Count	Sum	Average	Variance	*p*-Value	Pearson’s Correlation
1	ECSMS	1549	95,962.48	61.9512	1301.117	$6.83\times 10^{-9}$	0.956028
	Flexpoint	1549	81,151	52.3893	2892.75		
2	ECSMS	1283	79,945.22	62.3112	1321.545	$4.64\times 10^{-14}$ at 125°	0.826215 at 125°
	Fibre Optic	1283	107,135	83.5035	8695.534	$1.12\times 10^{-6}$ at 90°	0.950361 at 90°
3	ECSMS	1430	90,222.87	63.0929	1342.095	0.560302	0.999868
	IMU	1430	91,370.39	63.8954	1372.16		
4	ECSMS	1366	85,023.33	62.2426	1327.691	0.999559	1
	Hall effect	1366	85,022.27	62.2418	1327.688		

**Table 6:** ECSMS vs. sensor/glove technologies repeatability test results.

Summary							
Groups	Devices	Count	Sum	Average	Variance	*p*-Value	Pearson’s Correlation
1	ECSMS	460	25,998.09	59.08658	1273.547	0.708701687	0.93788
	Flexpoint	460	26,596	60.44545	4543.683		
2	ECSMS	460	28,646.61	62.27524	1395.011	$4.8182\times 10^{-7}$ at 125°	0.812248 at 125°
	Fibre Optic	460	40,600	88.26087	10,690.18	0.036 at 90°	0.917 at 90°
3	ECSMS	460	28,566.45	62.10098	1396.084	0.978330889	0.999944
	IMU	460	28,597.18	62.16778	1385.049		
4	ECSMS	460	26,222.44	59.59645	1272.426	0.999998329	0.999999
	Hall effect	460	26,222.44	59.59645	1272.916		

**Table 7:** ECSMS vs. Flexpoint repeatability test results.

ECSMS vs. Flexpoint						
Finger/Arm Angle and Method of Measurement	N	Min	Max	Mean	Range	SD
0.791099977	20	−2	0	−1	2	0.458
6.416699886	20	−3	0	−1.15	3	0.67
11.95440006	20	−2	0	−1	2	0.458
16.2614994	20	−2	0	−1	2	0.458
23.02979851	20	0	0	0	0	0
28.74329948	20	0	1	0.05	1	0.223
34.19309998	20	1	3	2.35	2	0.587
39.81869888	20	3	8	5.4	5	1.142
45.35639954	20	8	14	10.25	6	1.164
50.89410019	20	16	21	17.5	5	1.051
55.11330032	20	22	27	25.15	5	1.308
62.145298	20	36	48	39.55	12	2.35
67.85879517	20	48	60	54.15	12	2.433
73.57229614	20	61	79	67.15	18	3.407
79.46160126	20	74	87	79.45	13	2.999
85.08719635	20	87	107	97.55	20	5.02
90.80069733	20	109	119	112.7	10	2.25
96.33839417	20	120	138	129.55	18	4.058
101.9639969	20	142	166	151.75	24	5.793
107.5895996	20	150	187	169.2	37	8.924
113.0393982	20	173	223	187.75	50	10.119
118.577095	20	175	229	203.5	54	14.427
N = total number of measurements						
SD = standard deviation						

**Table 8:** ECSMS vs. fibre optic repeatability test results.

ECSMS vs. Fibre Optic						
Finger/Arm Angle and Method of Measurement	N	Min	Max	Mean	Range	SD
0.703199959	20	−14	6	−0.75	20	5.066
6.416699886	20	−2	19	7.2	21	6.048
11.8664999	20	−9	21	8.4	30	8.184
16.2614994	20	−6	20	11.25	26	6.987
22.94190025	20	−8	25	10.3	33	10.208
28.56749916	20	−2	25	12.8	27	8.569
34.10519791	20	11	39	26.2	28	7.345
39.81869888	20	11	45	24.85	34	9.258
45.35200444	20	5	51	33.15	46	10.168
50.89410019	20	23	66	45.65	43	11.15
56.5196991	20	38	75	56.3	37	11.216
62.05739975	20	35	86	64.05	51	16.433
67.77089691	20	38	104	62.6	66	17.475
73.48439789	20	39	97	75.2	58	17.431
79.37809066	20	47	110	73.55	63	14.391
84.9992981	20	65	112	85.4	47	15.567
90.71279907	20	71	116	93.6	45	13.468
96.25049591	20	66	145	110.7	79	21.203
101.8760986	20	92	181	136.55	89	22.333
107.4137955	20	121	230	168.45	109	28.858
112.7756958	20	150	316	201.35	166	44.959
118.3134003	20	192	391	304.15	199	59.567
N = total number of measurements						
SD = standard deviation						

**Table 9:** ECSMS vs. IMU repeatability test results.

ECSMS vs. IMU						
Finger/Arm Angle and Method of Measurement	N	Min	Max	Mean	Range	SD
0.88	20	0.96	1.15	1.0445	0.19	0.05
6.33	20	6.43	6.65	6.534	0.22	0.061
11.78	20	11.89	12.17	12.0365	0.28	0.088
17.392	20	17.45	17.81	17.6805	0.36	0.094
21.54	20	21.55	22.1	21.808	0.55	0.17
28.3	20	28.11	29.28	28.614	1.17	0.331
33.84	20	33.74	34.5	34.1685	0.76	0.26
39.56	20	39.43	40.2	39.828	0.77	0.281
44.92	20	44.66	45.51	45.0285	0.85	0.252
50.54	20	50.43	51.28	50.8305	0.85	0.324
56.143	20	55.85	56.98	56.3265	1.13	0.414
61.79	20	61.27	62.53	61.864	1.26	0.394
67.51	20	66.81	68.31	67.493	1.5	0.465
73.22	20	72.55	73.77	73.098	1.22	0.424
79.02	20	78.43	79.47	78.945	1.04	0.362
84.731	20	83.92	85.2	84.5515	1.28	0.43
90.54	20	89.36	91.15	90.301	1.79	0.586
96.07	20	95.24	96.67	95.839	1.43	0.524
101.79	20	100.8	102.98	101.6765	2.18	0.557
107.33	20	106.56	107.94	107.3845	1.38	0.488
112.9095	20	111.97	113.48	112.6695	1.51	0.492
118.3415	20	117.16	119.45	118.2595	2.29	0.628
N = total number of measurements						
SD = standard deviation						

**Table 10:** ECSMS vs. Hall effect repeatability test results.

ECSMS vs. Hall Effect						
Finger/Arm Angle and Method of Measurement	N	Min	Max	Mean	Range	SD
0.87800002	20	0.878	0.878	0.878	0	0
6.4094005	20	6.321602	6.321602	6.321602	0	0
11.940801	20	11.9408	11.9408	11.9408	0	0
17.560001	20	17.56	17.56	17.56	0	0
23.0914	20	23.0914	23.0914	23.0914	0	0
28.710602	20	28.7106	28.7106	28.7106	0	0
34.242001	20	34.242	34.242	34.242	0	0
39.773403	20	39.8612	39.8612	39.8612	0	0
45.304802	20	45.3048	45.3048	45.3048	0	0
50.924004	20	50.924	50.924	50.924	0	0
56.543201	20	56.5432	56.5432	56.5432	0	0
62.162403	20	62.1624	62.1624	62.1624	0	0
67.8694	20	67.8694	67.8694	67.8694	0	0
73.6642	20	73.6642	73.6642	73.6642	0	0
79.459	20	79.3712	79.3712	79.3712	0	0
84.99479195	20	84.9904	85.0782	84.99479	0.087799	0.019
90.697403	20	90.6974	90.6974	90.6974	0	0
96.228806	20	96.22881	96.22881	96.22881	0	0
101.84801	20	101.848	101.848	101.848	0	0
107.4672	20	107.4672	107.4672	107.4672	0	0
112.9108	20	112.9108	112.9108	112.9108	0	0
118.44221	20	118.53	118.53	118.53	0	0
N = total number of measurements						
SD = standard deviation						

## Data Availability

Not applicable.

## Supplementary Materials

The following are available online, Video S1: https://youtu.be/yFF0IhfUQFc (accessed on 24 February 2021).
